# A narrative systematic review of changes in mental health symptoms from before to during the COVID-19 pandemic

**DOI:** 10.1017/S0033291723002295

**Published:** 2023-08-24

**Authors:** Mary Blendermann, Tracie I Ebalu, Immanuela C Obisie-Orlu, Eiko I Fried, Lauren S Hallion

**Affiliations:** 1 Department of Psychology, University of Pittsburgh, Pittsburgh, PA, USA; 2 Department of Psychology, Leiden University, Leiden, The Netherlands

**Keywords:** COVID-19 pandemic, psychopathology, systematic review, anxiety, depression

## Abstract

The onset of the COVID-19 pandemic raised concerns regarding population-wide impacts on mental health. Existing work on the psychological impacts of disaster has identified the potential for multiple response trajectories, with resilience as likely as the development of chronic psychopathology. Early reviews of mental health during the pandemic suggested elevated prevalence rates of multiple forms of psychopathology, but were limited by largely cross-sectional approaches. We conducted a systematic review of studies that prospectively assessed pre- to peri-pandemic changes in symptoms of psychopathology to investigate potential mental health changes associated with the onset of the pandemic (PROSPERO #CRD42021255042). A total of 97 studies were included, covering symptom clusters including obsessive-compulsive disorder (OCD), post-traumatic stress disorder (PTSD), fear, anxiety, depression, and general distress. Changes in psychopathology symptoms varied by symptom dimension and sample characteristics. OCD, anxiety, depression, and general distress symptoms tended to increase from pre- to peri-pandemic. An increase in fear was limited to medically vulnerable participants, and findings for PTSD were mixed. Pre-existing mental health diagnoses unexpectedly were not associated with symptom exacerbation, except in the case of OCD. Young people generally showed the most marked symptom increases, although this pattern was reversed in some samples. Women in middle adulthood in particular demonstrated a considerable increase in anxiety and depression. We conclude that mental health responding during the pandemic varied as a function of both symptom cluster and sample characteristics. Variability in responding should therefore be a key consideration guiding future research and intervention.

As the financial, occupational, and social impacts of the COVID-19 pandemic became apparent, voices across clinical psychological science and medicine raised concerns regarding potential consequences for population-wide mental health (Fiorillo & Gorwood, 2020; Gruber et al., 2021; Pfefferbaum & North, 2020). Some early predictions anticipated a broad decline in mental health, including development of psychopathology in previously healthy individuals and exacerbated symptoms in clinical populations (Fiorillo & Gorwood, 2020; Gruber et al., 2021). Stress is a well-established risk factor for the development and worsening of psychopathology (Liu & Miller, 2014; McLaughlin, 2020), particularly internalizing symptoms such as anxiety and depression (Faravelli et al., 2012; Kendler & Gardner, 2016), and these predictions largely followed from conceptualizations of the COVID-19 pandemic as both an acute and chronic stressor (Bridgland et al., 2021; Waugh, Leslie-Miller, & Cole, 2022).

Other perspectives argued that mental health impacts of the COVID-19 pandemic would be more nuanced than population-level decline (Koushik, 2020; Mancini, 2020). For example, decreased work and social obligations might provide temporary relief from anxiety symptoms, while mandated social isolation might exacerbate depression (Koushik, 2020). An increased sense of togetherness, previously observed in communities experiencing mass trauma, could even benefit mental health (Lau et al., 2008; Mancini, 2020). Inter-individual differences may also play a role, with vulnerable populations shouldering the burden of worsening symptoms (Mancini, 2020). Such predictions are in line with prospective studies of the psychological impacts of disaster, with only a minority of participants (typically 30% or less) developing severe, chronic psychopathology (Bonanno, Brewin, Kaniasty, & Greca, 2010).

Early systematic reviews of general population mental health during the first few months of the COVID-19 pandemic reported startling rates of clinically significant anxiety (ranging from 26% to 38%), depression (26–34%), and psychological distress (30–38%; Deng et al. 2021; Krishnamoorthy, Nagarajan, Saya, & Menon, 2020; Luo, Guo, Yu, Jiang, & Wang, 2020; Necho, Tsehay, Birkie, Biset, & Tadesse, 2021; Salari et al. 2020). However, the studies covered by these reviews are largely epidemiological and cross-sectional, often with single item or unvalidated assessments. The absence of pre-pandemic timepoints in particular limits inferences about change over time that is potentially attributable to the pandemic. To our knowledge, only one meta-analysis has synthesized studies with a pre-pandemic timepoint (Robinson, Sutin, Daly, & Jones, 2022). Across 65 longitudinal cohort studies, there was a small overall increase in symptoms of psychopathology, with the largest symptom increases observed in studies that sampled participants early in the pandemic (March–April 2020). Although this meta-analysis focused on longitudinal cohort studies, samples were largely European and North American, and did not include symptom clusters beyond the broad categories of anxiety, depression, and psychological distress.

Important questions also remain regarding variability in trajectories across symptom types and populations. For example, OCD symptoms related to contamination and health behaviors (e.g. handwashing, disinfecting surfaces) may have been impacted differently than other kinds of internalizing symptoms (Guzick et al., 2021). PTSD symptoms might emerge in populations such as healthcare workers (Bridgland et al., 2021). Finally, theoretical and empirical frameworks distinguish between acute autonomic reactions to imminent threat (fear) *v*. prolonged apprehension involving chronic or distal threat (anxiety; Kotov et al., 2017; Öhman, 2008). Whereas fear might be expected to show a sharp increase relative to pre-pandemic followed by a steady decline, the evolving and prolonged nature of the pandemic suggests a potentially different course for anxiety. The present systematic review gives in-depth consideration to these and other internalizing-related symptom clusters. We adopt a global, lifespan, and transdiagnostic perspective, with findings reported separately for unselected samples, samples with diagnosed psychopathology, and selected samples such as patients with preexisting medical conditions or other special characteristics (e.g. veterans).

## Method

### Search strategy

A systematic review was conducted in accordance with PRISMA guidelines (Moher, Liberati, Tetzlaff, & Altman, 2009) and preregistered on PROSPERO (#CRD42021255042). We searched PubMed and PsycINFO for studies that assessed mental health symptoms both before and during the COVID-19 pandemic. Keywords included (COVID-19 OR coronavirus OR COVID OR pandemic OR SARS-CoV-2 OR social distancing OR quarantine OR lockdown) AND (anxi* OR obsess* OR trauma* OR fear OR panic OR agoraphobi* OR social anxiety OR separation anxiety OR acute stress* OR depress*). A database search was conducted from 29 January 2020 (the day before the World Health Organization designated the COVID-19 pandemic a Public Health Emergency of International Concern) to 24 June 2021. Complete search strategies for each database and deviations from the preregistration can be found in online Supplementary Materials.

#### Study selection

**Figure 1.**
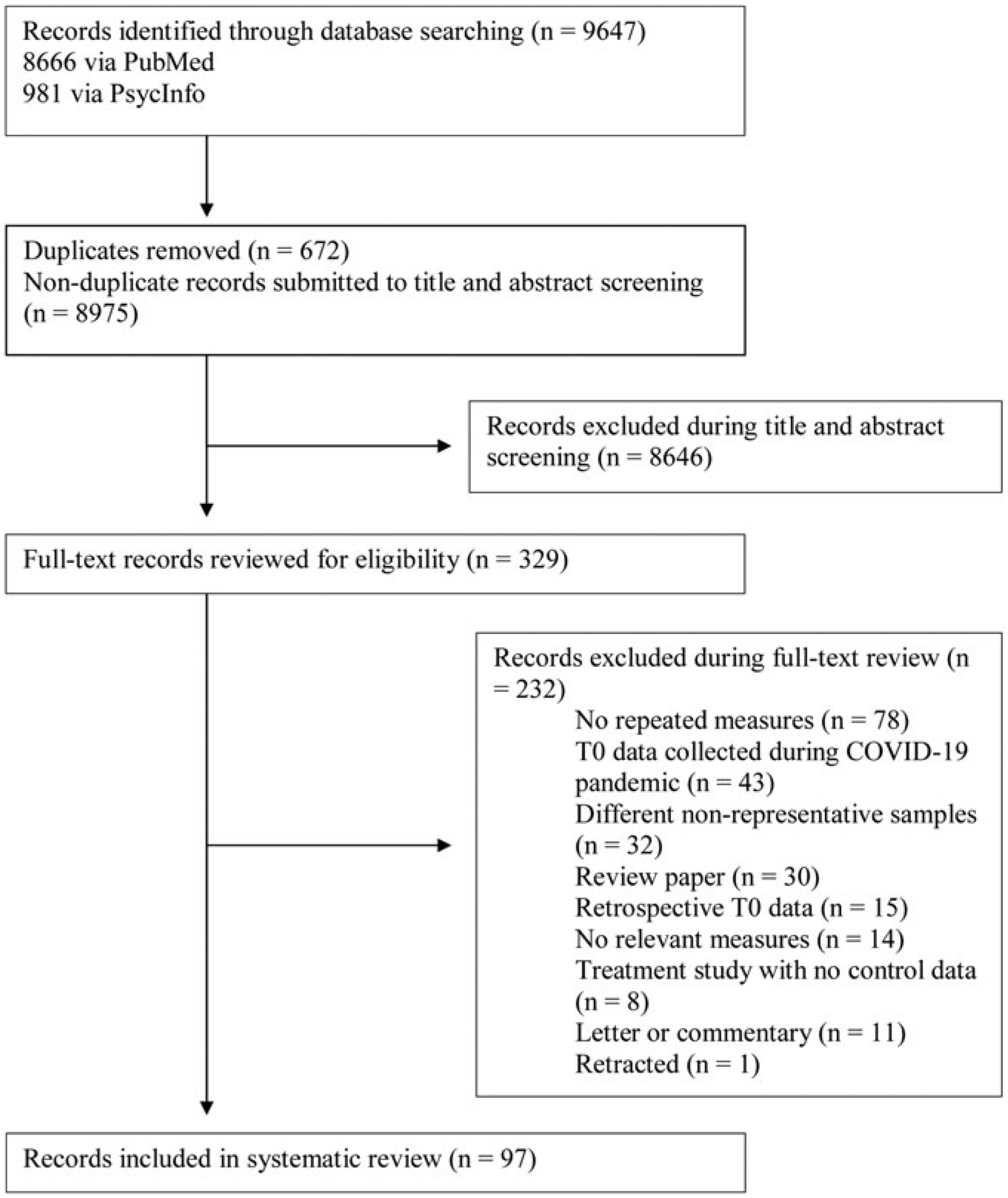
PRISMA diagram showing study selection process.

Titles and abstracts were screened according to eligibility criteria (outlined below). Full-text screening was conducted by four authors (M. B., T. I. E., I. C. O., and L. S. H.). Where eligibility was uncertain, a consensus decision was made following discussion. Study selection process and reasons for exclusion are described in Fig. 1.

##### Inclusion criteria.

Studies were included if they met all of the following criteria: (a) published or in press on or after 29 January 2020; (b) published in English; (c) participants and/or studies based in a country or region with confirmed COVID-19 cases, lockdown measures, and/or quarantine; and (d) assessed symptoms of mental health both prior to (no later than 29 January 2020) and during the COVID-19 pandemic, either in the same sample at both timepoints or in two nationally or regionally representative samples.

##### Exclusion criteria.

Studies were excluded if they met any of the following criteria: (a) cross-sectional design, or repeated measures in different, non-representative samples; (b) administered mental health treatment between the pre-pandemic and peri-pandemic assessments (healthy and no-treatment control groups remained eligible); (c) did not present original empirical work (e.g. commentaries, reviews).

##### Methodological quality.

Included studies were assessed for methodological quality by the first author across four domains: sample size, sampling methodology, response rate, and measurement (see online Supplementary Materials).

## Results

**Figure 2.**
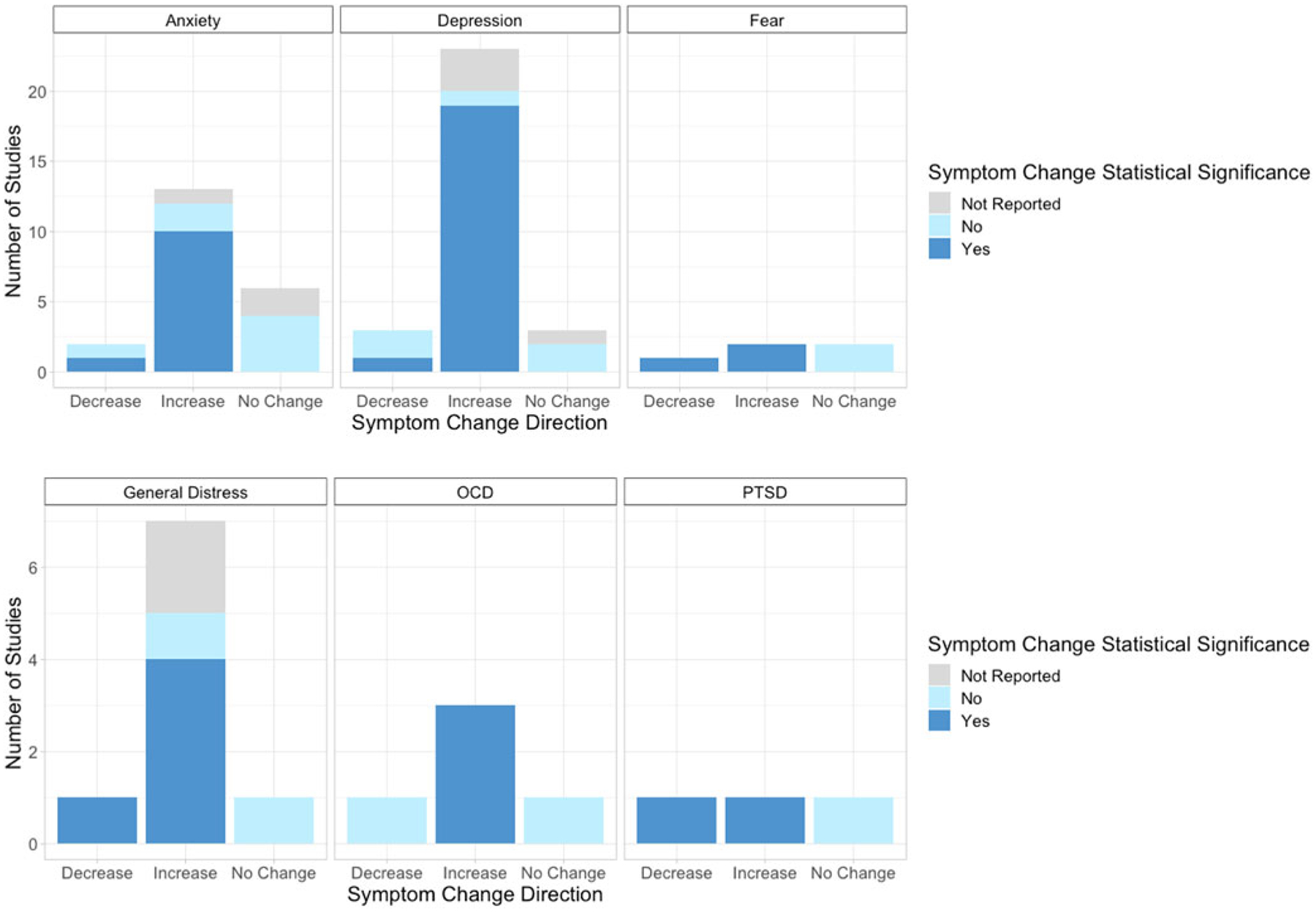
Distribution of studies in each symptom cluster reporting either an increase, decrease, or no change in symptoms. Colors reflect the reported statistical significance of each symptom change.

**Figure 3.**
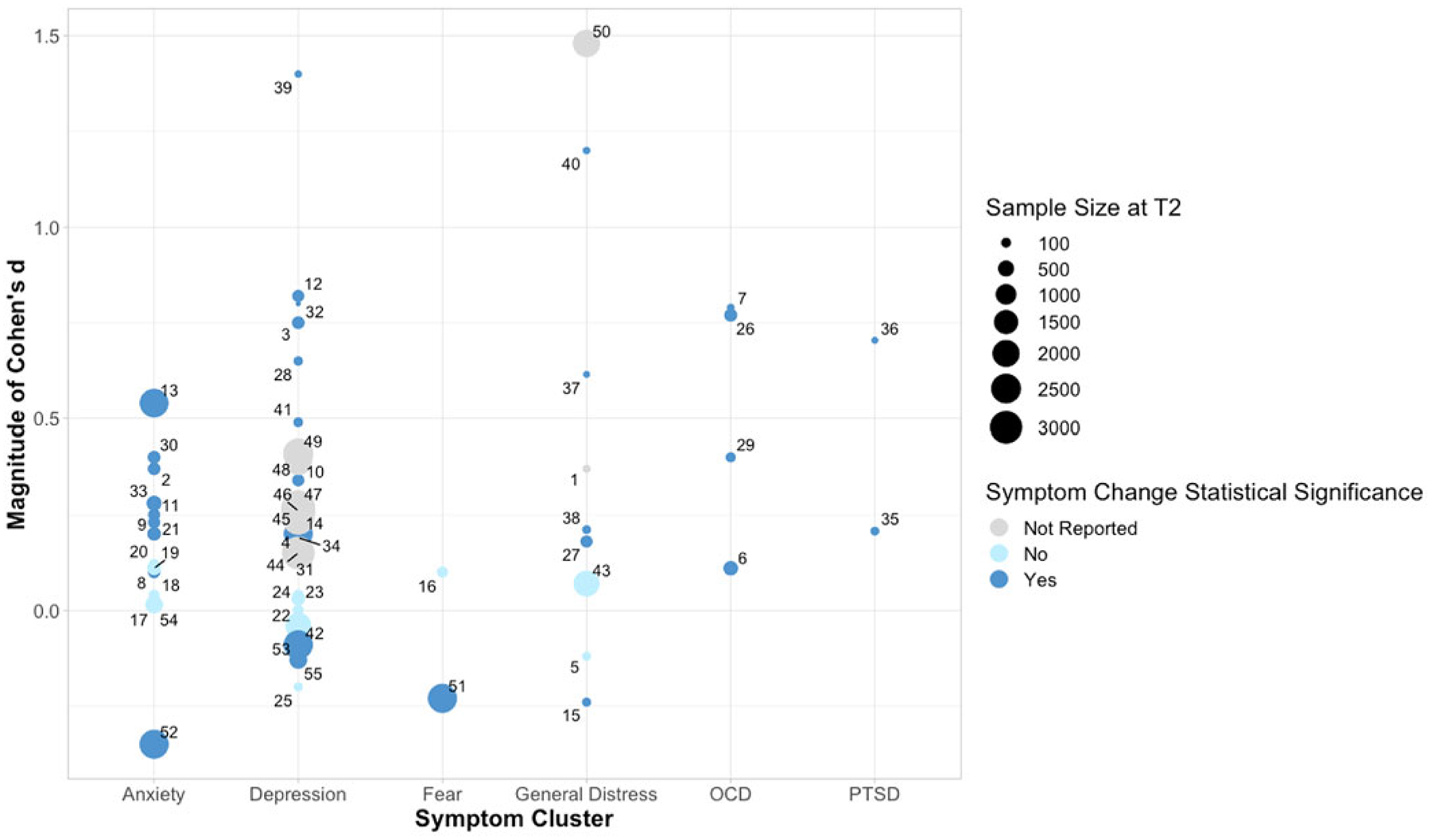
Spread of effect sizes for studies where Cohen’s *d* was reported or could be calculated (*k* = 55). Effect sizes <0 indicate symptom decrease; effect sizes >0 indicate symptom increase. See Supplementary Table S4 for studies corresponding to point labels.

The search terms yielded 981 results from PsycINFO and 8666 results from PubMed, for a total of 9647 potentially eligible items. Following the removal of 672 duplicate items, 8646 items were excluded during title and abstract screening, and 232 items were excluded during full-text screening, yielding 97 studies which were included in the final review (see Figs 2–3 and Table 1 for included studies). Symptom changes described below were statistically significant unless otherwise specified.

### Obsessive-compulsive disorder (*k* = 11)

Three of four studies in unselected (including undergraduate student) samples reported increases in overall OCD symptoms, particularly washing and contamination symptoms (Cox & Olatunji, 2021; Jelinek, Göritz, Miegel, Moritz, & Kriston, 2021; Knowles & Olatunji, 2021). The fourth, a longitudinal cohort study of 2117 Brazilian university employees, found no change in the prevalence of OCD diagnoses from pre- to peri-pandemic (Brunoni et al., 2021). Of five studies including participants with pre-existing OCD diagnoses (*N* = 60 to *N* = 270), four found that OCD severity increased. In one study of an outpatient clinic sample, pre-existing contamination symptoms predicted increases in overall OCD severity (Davide et al., 2020). A study of children and adolescents with OCD treated at a university psychiatry department only found changes in washing and contamination symptoms (Tanir et al., 2020). In a Spanish sample of 127 clinic outpatients with OCD, ~31% showed at least a moderate increase in severity (over 25%), with the remaining changes ranging from a small increase to a small decrease (Alonso et al., 2021). Similarly, about half of a sample of 84 Indian adults with OCD showed no change, while the other half mostly showed increases in severity of less than 5% (Chakraborty & Karmakar, 2020). In samples with psychiatric diagnoses other than OCD, a study of 80 children and adolescents with various neurologic and psychiatric disorders found an increase in OCD symptoms (Conti et al., 2020), while a study of 35 Catalán adults with autism found no changes (Lugo-Marín et al., 2021).

Across samples, OCD symptoms tended to increase, with few exceptions. Calculable effect sizes (*k* = 4) were small to moderate (median Cohen’s *d* = 0.59, range = 0.11–0.79). Consistent with predictions, symptom increase was more pronounced for washing-checking domains and in diagnosed samples.

### Post-traumatic stress disorder (*k* = 5)

Findings for PTSD were inconclusive, driven in part by the small total number of studies. The largest study available, in a nationally representative panel survey of 3078 American veterans, did not find significant changes in PTSD probable diagnosis prevalence (Hill et al., 2021). In a sample of 473 Canadian adults, no changes in PTSD symptoms were identified (Minhas et al., 2021). An increase in PTSD symptoms was observed in 80 Italian children and adolescents with a pre-existing neuro-developmental disorder (Conti et al., 2020), as well as in 85 German adults with and without psychiatric disorders (Seitz, Bertsch, & Herpertz, 2021). By contrast, there was a decrease in PTSD severity among 76 older adults with a pre-existing PTSD diagnosis compared to trauma-exposed controls in the USA (Rutherford et al., 2021).

### Fear (*k* = 10)

Six studies assessed fear/autonomic anxiety in unselected samples, with variable results. A study of 217 Indian undergraduates found a small increase in fear (Saraswathi et al., 2020), as did two smaller studies [99 adult women in Poland (Ilgen, Kurt, Aydin, Bilen, & Kula, 2021) and 68 Italian undergraduates (Bussone, Pesca, Tambelli, & Carola, 2020)]. By contrast, a study of 2364 Chinese undergraduates found a slight decrease in fear (Yang, Ji, et al., 2021), as did a study of 66 Brazilian pharmacy students (Campos, Campos, Bueno, & Martins, 2021). The remaining study, in a crowdsourced sample of 146 American adults, found no change (Haliwa, Wilson, Lee, & Shook, 2021).

Both studies including samples with pre-existing psychiatric diagnoses found no change in fear [275 American adults with autism spectrum disorder drawn from an existing research registry (Adams, Zheng, Taylor, & Bishop, 2021); 1181 adults with internalizing disorders drawn from a prospective longitudinal study in the Netherlands (Pan et al., 2021)]. The latter study did report a small increase in fear among 336 healthy control participants (recruited through primary care settings; Pan et al., 2021). Two studies of selected samples showed stronger effects, including a clinically significant increase in fear in 595 Turkish cancer patients (Yildirim, Poyraz, & Erdur, 2021), and an increase in the proportion of 63 pregnant participants meeting the threshold for moderate or severe fear (Ayaz et al., 2020).

Taken together, findings suggest that medically selected samples experienced a clinically significant increase in fear, but this pattern did not apply to unselected samples or those with pre-existing psychiatric vulnerabilities, who tended to experience at most small increases. Although several studies reporting null results had small sample sizes, a few larger studies also showed no change in fear severity, suggesting that null results are unlikely to be due to low statistical power.

### Anxiety (*k* = 47)

Of 21 studies prospectively examining anxiety in unselected samples, 11 found an increase in anxiety and eight found no change, while two studies reported a decrease. One of the largest studies, a randomly sampled cohort study of 113 928 German adults ages 20–74, reported an increase in anxiety for participants under 60 years of age only, with women ages 20–39 showing the largest increase (Peters, Rospleszcz, Greiser, Dallavalle, & Berger, 2020). In a prospective cohort study of 1237 French adults (Ramiz et al., 2021), the proportion of participants with ‘possible anxiety’ (GAD-7 score >4) increased over time, with the most marked increases occurring for women ages 23–49 and ages 70 or above. A study comparing two randomly sampled, nationally representative samples of Czech adults (Winkler et al., 2021) similarly found that the prevalence of anxiety disorders increased from 8% in November 2017 to 13% in May 2020. Younger adults, women, those who struggled to retain employment, and those without a high school diploma had the highest absolute rates of anxiety disorder during the second wave of the pandemic.

Well-powered studies that did not find changes in anxiety include a representative sample of 1041 Irish adults (Hyland et al., 2021) and a nationally representative study of 944 604 American adults compared to a 2019 propensity-matched sample (Jacobs & Burch, 2021). However, interpretability of these large studies may be offset by other methodological constraints (use of a cut-off score and single-item assessment, respectively).

The only two studies reporting a decrease in anxiety were a study of 2364 Chinese undergraduates (assessed October 2019, February 2020, and May 2020; Yang, Ji., et al., 2021) and 2117 Brazilian adults (May–July 2020 compared to 2008–2010, with no change compared to 2016–2018; Brunoni et al., 2021).

Five studies examined anxiety in psychiatric samples. One study (Pan et al., 2021) found an increase in worry across 1181 adults with psychiatric illness and 336 healthy controls from three longitudinal cohort studies in the Netherlands. Another longitudinal cohort study found an increase in 147 healthy controls, but *not* 345 adults with bipolar disorder (Yocum, Zhai, McInnis, & Han, 2021). Two smaller studies also found an increase in anxiety; in 76 Chinese participants receiving methadone maintenance treatment for substance use disorder (Liu et al., 2021); and in 46 American older adults with PTSD, but not 30 trauma-exposed controls (Rutherford et al., 2021). A third study found no change in a sample of 35 Catalán adults with autism (Lugo-Marín et al., 2021).

Seven studies prospectively assessed changes in anxiety in children and adolescents with remarkably consistent findings; all but one found at least a small increase in anxiety coinciding with the onset of the pandemic (Breaux et al., 2021; Conti et al., 2020; Magson et al., 2021; Rogers, Ha, & Ockey, 2021). Studies reporting an increase in anxiety include a sample of 775 children and adolescents assessed via an experience-sampling application in Australia (Arjmand, Seabrook, Bakker, & Rickard, 2021); a representative sample of 844 Dutch children and adolescents (compared to a representative sample from 2018; Luijten et al., 2021); and 136 Canadian children and adolescents (De France, Hancock, Stack, Serbin, & Hollenstein, 2021), who demonstrated a marginal increase in anxiety that was driven by an increase in girls only.

Ten studies prospectively assessed changes in anxiety in older adults and other medically vulnerable samples. Relatively larger studies tended to find an increase in anxiety, including a multi-national sample of 435 adults with systemic sclerosis recruited during medical visits (Thombs et al., 2020); 538 Chinese older adults with two or more chronic health conditions (Wong et al., 2020); 721 Chilean older adults from a random community sample (Herrera et al., 2021); and 133 American adults with HIV (but not 54 healthy controls; Cooley, Nelson, Doyle, Rosenow, & Ances, 2021). Null findings were observed in a prospective observational study of 1051 patients with remitted breast cancer (Mink van der Molen et al., 2021); 450 Australian adults with type 2 diabetes (Sacre et al., 2021); and 411 Chinese older adults (Siew, Mahendran, & Yu, 2021).

Studies of other selected samples also tended to find increases in anxiety. A longitudinal cohort study of 2288 American sexual and gender minority (SGM) adults found an increase in anxiety severity during the early stages of the pandemic (Flentje et al., 2020). However, this increase was driven by participants who were relatively lower in anxiety, with those who screened positive for GAD prior to the pandemic showing no change. A study of 1028 recent mothers in a hospital-based birth cohort in Brazil found a twofold increase in GAD prevalence from 2019 (during pregnancy) to 2020 (Loret de Mola et al., 2021). A nationally representative study of 3078 predominantly male American veterans found an increase in the prevalence of positive GAD screenings from 7% to 9%, driven by a marked increase in anxiety severity among middle-aged veterans (Hill et al., 2021).

Collectively, most studies found an increase in anxiety associated with the onset of the pandemic, although calculable effect sizes (*k* = 14) tended to be small (median Cohen’s *d* = 0.16, range = −0.35 to 0.54). Findings were especially pronounced and consistent in child and adolescent samples and in medically vulnerable participants. Psychiatric samples also showed some vulnerability to an increase in anxiety, but this vulnerability was not more pronounced than that observed in unselected samples.

### Depression (*k* = 74)

Twenty-seven studies prospectively assessed changes in incidence or severity of depression in unselected samples. Only two studies found a decrease in severity, one in 1020 Irish adults (Hyland et al., 2021) and one in 2364 Chinese undergraduate students (Yang, Ji, et al., 2021). The remaining studies found either an increase (*k* = 17) or no change (*k* = 8) in severity. In the largest study (*N* = 113 928 German adults; Peters et al., 2020), incidence of moderate-to-severe depression symptoms increased from 6.4% 1–5 years before the pandemic to 8.8% in May 2020. Incidence of depression increased by 7.8% (from 4% in November 2017 to 11.8% in May 2020) across two nationally representative samples of over 3000 Czech adults (Winkler et al., 2021). Incidence doubled across two representative random samples of Chinese adults (from 6.3% of 4054 adults in 2017 to 14.8% of 1501 adults in April 2020; Zhao et al., 2020); and increased more than fivefold in a representative random sample of 715 Czech adults (Novotný et al., 2020).

Studies comparing demographic groups found that younger adults (e.g. under age 60; Peters et al., 2020) and women (Fruehwirth, Biswas, & Perreira, 2021; Minhas et al. 2021; Peters et al. 2020) were especially vulnerable to increased depression incidence or severity. Studies of undergraduates had proportionately more non-significant results, which may be due in part to smaller sample sizes (although see findings from Yang, Ji, et al., 2021, above).

Of seven studies including psychiatric samples, findings were mixed, with increases in depression severity reported in a sample of 1181 Dutch participants with internalizing disorders and in 336 healthy controls (Pan et al., 2021); 52 German adults with eating disorders (Giel, Schurr, Zipfel, Junne, & Schag, 2021); and 76 Chinese adults with substance use disorder (Liu et al., 2021). The remaining studies reported no change in depression in 275 American adults with autism (Adams et al., 2021); a decrease in 35 Catalán adults with autism (Lugo-Marín et al., 2021); or non-significant changes (Rutherford et al., 2021; Yocum et al., 2021).

Of nine studies with child and adolescent samples, all but two reported an increase in depression severity, with some studies reporting small effect sizes (e.g. Magson et al., 2021; Rogers et al., 2021), and others reporting moderate-to-large effects (e.g. Breaux et al., 2021; Thorisdottir et al., 2021). Only one large study did not find changes (1778 Chinese children and adolescents recruited through school-based cluster sampling; Teng, Pontes, Nie, Griffiths, & Guo, 2021). Studies that examined demographic predictors tended to find worse outcomes for girls compared to boys (e.g. De France et al., 2021; Thorisdottir et al., 2021) and for children whose parents experienced employment difficulties (e.g. Luijten et al., 2021).

Of seven studies examining depression in older adults, five reported an increase in incidence or severity. In the largest study, a national opt-in panel survey of 16 644 older American adults (Barcellos, Jacobson, & Stone, 2021), this increase was driven by an increase in women only. Of 13 studies examining changes in depression in other medically vulnerable individuals, seven reported increases in severity, though only two studies with sample sizes >100 found this increase. The remainder of larger studies reported no change (*k* = 4) or a decrease (*k* = 1). This pattern is potentially suggestive of a true null effect.

Seven studies assessed other selected samples, with all but the smallest reporting an increase in incidence or severity of depression, typically with moderate or large effect sizes. A study of 2288 American SGM individuals found a small increase in self-reported depression severity (Flentje et al., 2020). In a sample of 419 American undergraduates (Fruehwirth et al., 2021), increased depression was observed in both SGM and non-SGM students. Four studies assessed participants who were pregnant or newly post-partum, with *N*s ranging from 50 (Lorentz et al., 2021) to 1042 (Loret de Mola et al., 2021).

Across studies, findings for depression did not follow a clear pattern. While the majority of studies reported increased depression incidence or severity, other studies in similar populations found no change or a decrease. Effect sizes (*k* = 24) tended to be small (median Cohen’s *d* = 0.22, range = −0.2 to 1.4).

### General distress (*k* = 23)

Of 10 studies investigating general psychological distress in unselected samples, seven reported pandemic-related increases. These included the three largest studies, including two nationally representative samples (2032 American adults, Twenge & Joiner, 2020; 13 754 adults in the UK, Chandola, Kumari, Booker, & Benzeval, 2020). The magnitude of these increases varied, but tended to be large. Only two studies found a decrease in general distress, in 555 Chinese undergraduate students (Li, Cao, Leung, & Mak, 2020); and 71 Brazilian undergraduates (da Freitas, de Medeiros, & de Lopes, 2021). In both cases, absolute severity was low at both occasions.

Of four studies examining general distress in psychiatric samples, increased severity was observed in 76 Chinese adults undergoing methadone maintenance treatment (Liu et al., 2021); 32 children with dyslexia and their mothers (Soriano-Ferrer, Morte-Soriano, Begeny, & Piedra-Martínez, 2021); and 66 adults with psychiatric diagnoses and 22 healthy controls (Seitz et al., 2021). Only one study, of 37 children and 35 adults on the autism spectrum (Lugo-Marín et al., 2021), found no change in psychiatric distress.

Results for children and adolescents without psychiatric diagnoses were variable. A study of 1778 Chinese youth who play video games (Teng et al., 2021) found a small increase in distress. However, a study of 127 Canadian youth found a decrease (Gagné, Piché, Clément, & Villatte, 2021), while a study of 203 Dutch youth found no change (Achterberg, Dobbelaar, Boer, & Crone, 2021).

Of six studies in medically vulnerable samples, four found an increase in distress (including both studies in pregnant women; Ayaz et al., 2020; Perzow et al., 2021), while the remainder found no change. Small sample size constrains interpretability for these studies, as the largest (177 dialysis patients; Bonenkamp et al., 2021) found no change in distress, while the next largest (135 pregnant participants; Perzow et al., 2021) found an increase. The remaining studies had fewer than 70 participants each and were likely underpowered to detect increases of small magnitude. Finally, a longitudinal cohort study of 208 American transgender and gender non-binary individuals found an increase in distress (Kidd et al., 2021).

There was a stable tendency for distress to increase in adult samples. Calculable effect sizes (*k* = 10) tended to be small (median Cohen’s *d* = 0.29; range = −0.24 to 3.8). Undergraduate students and children demonstrated the most consistent exception to this pattern, often showing a decrease in general distress.

## General discussion

The present systematic review found that changes in psychopathology from pre- to peri-pandemic varied as a function of symptom cluster and sample characteristics. Contrary to expectations (e.g. Gruber et al., 2021), adults with pre-existing mental health conditions were not disproportionately affected, excepting adults with pre-existing OCD, whose symptoms tended to worsen. Age also showed unexpected effects. Several large studies (Peters et al., 2020; Ramiz et al., 2021; Winkler et al., 2021) found more striking increases in anxiety symptoms in children and relatively younger adults, despite those being among the demographics least susceptible to serious COVID-19 infection. Studies of older adults and medically vulnerable individuals tended to have relatively smaller samples and more mixed results, though studies with larger samples found increases in anxiety and fear. Symptom trajectories were similarly variable; OCD and distress-related psychopathology (anxiety; depression) tended to increase, while PTSD and fear-related psychopathology failed to show a consistent pattern.

These patterns are likely multidetermined, but some candidate explanations can be offered. Different trajectories for anxiety (generally increased) *v*. fear (generally remained stable) may be attributable in part to the time course of the pandemic. Because acute fear reactions unfold on a much shorter timeline compared to anxiety, assessments weeks or months into the pandemic may have captured increased anxiety, but missed an initial uptick in fear. That women in their 20s–40s showed an especially prominent increase in anxiety and depression stands in contrast to their lower medical risk, and aligns with empirical findings that women took on more caregiving work than men when schools and childcare facilities closed during lockdown (OECD, 2021), which may have been especially stressful in light of reduced social support. Samples with pre-existing psychopathology tended to show increases in OCD symptoms, anxiety, and general distress, perhaps due to heightened vulnerability to contamination fears and prolonged uncertainty associated with a viral pandemic. Student samples, individuals with autism, and medical samples were the most likely to demonstrate stable or decreasing symptoms, suggesting that pandemic-related reductions in academic and social demands may have actually reduced overall stress for these populations.

The conclusions of the present review should be interpreted in light of its relative strengths and limitations. The reviewed studies varied in quality, with tradeoffs evident. The largest and most representative studies tended to use briefer assessments, and countries were not evenly represented. Methodological quality coding indicated that most studies used validated measures and reported average to good response rates, but often relied on smaller convenience samples. This suggests that while samples were characterized accurately, they may have failed to include those less likely to participate in voluntary mental health research, such as older individuals or those with existing psychopathology. However, many of the reviewed studies targeted samples with vulnerabilities related to age and psychiatric or medical characteristics, potentially mitigating bias introduced by non-random sampling methods. Although formal meta-analysis or modeling symptom trajectories was not possible due to considerable variability in utilized measures and reporting standards, we placed greater interpretative weight on studies with larger samples and well-validated assessments (and see Table 1, which includes sample nationality and months elapsed between assessments).

Taken together, these findings underscore the importance of specificity in investigating and responding to pandemic-related changes in mental health. Findings are more consistent with theoretical conceptualizations of the pandemic as a chronic stressor, *v*. an acute trauma. Increased mental health symptoms may have reflected contextual adaptations to a high-risk environment (i.e. ‘true alarms’). Future studies of mental health in disaster contexts should consider the functional context (e.g. potential adaptive value) and impairment associated with symptom changes, while carefully weighing psychometric considerations, such as assuming measurement invariance. Demographic factors such as age, gender, socioeconomic status, and marginalized identity status should also be assessed as possible hidden moderators of disaster impact on mental health. In the present review, although lifespan risk factors such as pregnancy and older age were associated with increased internalizing symptoms, medically vulnerable and most psychiatric populations showed unexpected resilience, which suggests the potential value of a strengths-based perspective.

Anxiety and other forms of internalizing psychopathology have long been conceptualized as evolutionary adaptations that operate in excess in modern, generally safe contexts (Öhman & Mineka, 2001). Chronically anxious individuals may have experienced a sense of validation from the societal consensus that the environment was unsafe, or may have been more experienced in navigating day-to-day life while anxious. This resilience was not universal, however. Individuals with contamination-related OCD in particular experienced a worsening of symptoms, perhaps due in part to public health messaging around risks of the virus and responsibility for preventing harm. From a clinical and public health perspective, children and relatively younger adults, and particularly younger women, appear to be shouldering most of the mental health burden. Additional research is needed to identify the major psychological determinants of these vulnerabilities (e.g. caregiving responsibilities; social isolation) to best inform the development of public policy and interventions.

## Supplementary Material

supplemental materials

## Figures and Tables

**Table 1.** Summary of included studies, sample characteristics, and symptom change results

Author (year)	Country	T1 data collection started	Months elapsed T1–T2	Response rate (%)	T1 *N*	T2 *N*	Sample age group	Age (*M*)	Sample characteristics	Symptom measure	Statistical approach	Symptom change direction	*p* value
Obsessive-compulsive disorder													
Unselected and undergraduate samples													
Brunoni et al. (2021)	Brazil	2008	14	51.7	2117	-	Adults	62.32	-	CIS-R	Cochran’s Q test for paired data	Decrease	ns
Cox and Olatunji (2021)	USA	2016	39	29	369	369	Adults	46.98	-	OCI-R	Paired *t* test	Increase	0.04
Jelinek et al. (2021)	Germany	3/30/2014	71	90.5	538	1207	Adults	55.83	-	OCI-R	Repeated-measures ANOVA	Increase	<0.001
Knowles and Olatunji (2021)	USA	01/2020	1	-	108	108	Adults	19.62	Undergraduate students	Padua Inventory Contamination Subscale, OCI-R	Paired *t* test	Increase	<0.001
Psychiatric samples													
Alonso et al. (2021)	Spain	12/14/2019	1	-	127	127	Adults	42	OCD	YBOCS	*t*-test	Increase	<0.001
Chakraborty and Karmakar (2020)	India	-	-	-	84	84	Adults	-	OCD	YBOCS	Percentage score change	-	-
Davide et al. (2020)	Italy	01/2020	1.5	-	30	30	Adults	43.17	OCD	YBOCS	Paired *t* test	Increase	<0.001
Khosravani, Aardema, Samimi Ardestani, and Sharifi Bastan (2021)	Iran	-	-	35.3	270	270	Adults	36	OCD	YBOCS	Paired *t* test	Increase	<0.001
Lugo-Marín et al. (2021)	Spain (Catalonia)	-	-	-	35	35	Adults	32.8	Autism spectrum disorder	SCL-90-R	Wilcoxon signed-rank test	Decrease	-
Child and adolescent psychiatric samples													
Conti et al. (2020)	Italy	-	7	-	80	80	Children and adolescents	-	Neurological and psychiatric disorders	CBCL 6–18	Paired *t* test	Increase	<0.05
Tanir et al. (2020)	Turkey	09/2019	1	67.8	61	61	Children and adolescents	13.62	OCD	CY-BOCS	Wilcoxon signed-rank test	Increase	<0.001
Post-traumatic stress disorder													
Conti et al. (2020)	Italy	-	7	-	80	80	Children and adolescents	-	Neurological and psychiatric disorders	CBCL 6–18	Paired *t* test	Increase	<0.1
Hill et al. (2021)	USA		7	75.6	4069	3078	Older adults	63.2	Veterans	PCL-5	McNemar’s test	-	0.14
Minhas et al. (2021)	Canada	-	-	73	473	473	Adults	23.8	-	PCL-5	Linear mixed-effects models	-	0.66
Rutherford et al. (2021)	USA	-	1	-	30	30	Older adults	67.4	Trauma exposed comparison group	PCL-5	Generalized linear mixed models	-	0.738
Rutherford et al. (2021)	USA	-	1	-	46	46	Older adults	62.5	PTSD	PCL-5	Generalized linear mixed models	Decrease	0.0008
Seitz et al. (2021)	Germany	09/2018	5	60.7	22	22	Adults	31.3	Healthy volunteers	PCL-5	Paired *t* test	Increase	-
Seitz et al. (2021)	Germany	09/2018	5	60.7	63	63	Adults	31.3	MDD, PTSD, or somatic symptom disorder	PCL-5	Paired *t* test	Increase	-
Fear													
Unselected and undergraduate samples													
Campos et al. (2021)		08/2019	6	22.4	294	66	Adults	21.7	Pharmacy students	DASS-21 Anxiety subscale	Prevalence rates	Decrease	-
Bussone et al. (2020)	USA	-	6	-	68	68	Adults	-	Undergraduate students	SCL-90-R Anxiety subscale	Repeated-measures ANOVA	Increase	0.03
Haliwa et al. (2021)		09/2019	3	36	146	146	Adults	43.75	MTurk sample (2 of 3)	DASS-21 Anxiety subscale	Paired *t* test	-	0.23
Ilgen et al. (2021)	Poland	-	-	-	99	99	Adults	35	-	BAI	Paired *t* test	Increase	<0.01
Pan et al. (2021)	The Netherlands	2006	39	58	336	336	Adults	57.7	Healthy controls	BAI	Mixed-effects models	Increase	0.032
Saraswathi et al. (2020)	India	12/2019	6	90.8	217	217	Adults	20	Undergraduate students	DASS-21 Anxiety subscale	Wilcoxon signed-rank test	Increase	<0.001
Yang, Ji, et al. (2021)	China	10/2019	4	-	2364	2364	Adults	20.4	Undergraduate students	DASS-21 Anxiety subscale	Paired *t* test	Decrease	<0.001
Psychiatric samples													
Adams et al. (2021)	USA	3/11/2020	2	87.3	275	275	Adults	26.45	Autism spectrum disorder	DASS-21 Anxiety subscale	Repeated-measures ANCOVA	-	ns
Pan et al. (2021)	The Netherlands	2006	39	58	1181	1181	Adults	56	Depression, anxiety, or OCD	BAI	Mixed-effects models	-	range, 0.062–0.44
Pan et al. (2021)	The Netherlands	2006	39	58	1181	1181	Adults	56	Depression, anxiety, or OCD	BAI	Mixed-effects models	-	range, 0.062–0.44
Other selected samples													
Ayaz et al. (2020)	Turkey	-	-	-	63	63	Adults	30.35	Pregnant women	BAI	-	Increase	0.004
Yildirim et al. (2021)	Turkey	02/03/2020	1	93.4	595	595	Adults	50.48	Breast, ovarian, colorectal, or gastric cancer patients	BAI	-	Increase	-
Anxiety													
Unselected and undergraduate samples													
Algattas, Roy, Agarwal, and Maroon (2021)	USA	1/1/2020	1	89.5	19	17	Adults	-	Neurosurgery residents	PSS	Paired *t* test	Decrease	0.515
Baiano, Zappullo, Group, and Conson (2020)	Italy	11/4/2019	2	80.6	25	25	Adults	23.84	-	PSWQ	Two-tailed Mann-Whitney *U* test	-	>0.05
Brunoni et al. (2021)	Brazil	2008	14	51.7	2117	-	Adults	62.32	-	CIS-R	Cochran’s *Q* test for paired data	Decrease	<0.001
Campos et al. (2021)	Brazil	08/2019	6	22.4	294	66	Adults	21.7	Pharmacy students	DASS-21 Stress subscale	Prevalence rates	-	-
Cooley et al. (2021)	USA	-	-	-	54	54	Adults	48.5	-	HADS Anxiety subscale	Mean difference	Increase	-
Daly and Robinson (2021)	USA	2019	3	-	30 915	6813	Adults	-	-	GAD-2	Logistic regression	-	<0.001
Elmer, Mepham, and Stadtfeld (2020)	Switzerland	2018	7	-	212	212	Adults	-	Undergraduate students	GAD-7	Paired *t* test	Increase	0.014
Evans, Alkan, Bhangoo, Tenenbaum, and Ng-Knight (2021)	UK	10/2019	5	84.1	251	251	Adults	19.76	Undergraduate students	HADS	Repeated-measures ANOVA	-	0.782
Feinberg et al. (2021)	USA	2017	3	53.6	206	206	Adults	-	Parents	PSWQ	Hierarchical linear modeling	Increase	0.044
Fruehwirth et al. (2021)	USA	1/2020	4	42	419	419	Adults	18.9	Undergraduate students	GAD-7	Prevalence rates	-	<0.05
Haliwa et al. (2021)		09/2019	3	35	142	142	Adults	40.46	MTurk sample (3 of 3)	PSS	Paired *t* test	-	0.91
Haliwa et al. (2021)		09/2019	3	35	142	142	Adults	40.46	MTurk sample (3 of 3)	GAD-7	Paired *t* test	-	0.62
Haliwa et al. (2021)		09/2019	3	58	300	300	Adults	41.38	MTurk sample (1 of 3)	PSS	Paired *t* test	-	0.06
Haliwa et al. (2021)		09/2019	3	36	146	146	Adults	43.75	MTurk sample (2 of 3)	DASS-21 Stress subscale	Paired *t* test	-	0.17
Haliwa et al. (2021)		09/2019	3	58	300	300	Adults	41.38	MTurk sample (1 of 3)	GAD-7	Paired *t* test	-	0.001
Hyland et al. (2021)	Ireland	02/2019	13	-	1020	1041	Adults	44.04	-	GAD-7	Structural equation modeling	-	0.208
Jacobs and Burch (2021)	USA	2019	4	-	944 604	944 604	Adults	51.14	-	Custom item	No statistical comparison	-	-
Lau, Shariff, and Meehan (2021)	England	-	3	-	104	104	Adults	-	Undergraduate students	GAD-7	Paired *t* test	Increase	ns
Lee, Cadigan, and Rhew (2020)	USA	01/2020	3	95	564	564	Adults	25.1	-	PHQ-4	Count ratios	Decrease	0.386
Minhas et al. (2021)	Canada	-	-	73	473	473	Adults	23.8	-	GAD-7	Linear mixed-effects models	Increase	<0.001
Novotný et al. (2020)	Czech Republic	-	-	39.2	715	715	Adults	46.12	-	PSS	Wilcoxon signed-rank test	Increase	<0.001
Pan et al. (2021)	The Netherlands	2006	39	58	336	336	Adults	57.7	Healthy controls	PSWQ	Mixed-effects models	Increase	<0.0001
Peters et al. (2020)	Germany	-	1	55.5	113 928	113 928	Adults	50	-	GAD-7	-	Increase	-
Ramiz et al. (2021)	France	2014	3.5	12.8	1237	1237	Adults	62	-	GAD-7	-	Increase	-
Saraswathi et al. (2020)	India	12/2019	6	90.8	217	217	Adults	20	Undergraduate students	DASS-21 Stress subscale	Wilcoxon signed-rank test	Increase	<0.001
Winkler et al. (2021)	Czech Republic	2017	30	-	3306	3021	Adults	47.87	-	MINI	-	-	-
Yang, Ji, et al. (2021)	China	10/2019	4	-	2364	2364	Adults	20.4	Undergraduate students	DASS-21 Stress subscale	Paired *t* test	Decrease	<0.001
Yocum et al. (2021)	USA	-	12	62	147	147	Adults	49	-	GAD-7	Generalized estimating equations	Decrease	<0.0001
Zhang, Zaman, Silenzio, Kautz, and Hoque (2020)		01/2020	-	100	49	49	Adults	-	Undergraduate students	GAD-7	-	-	-
Zhao et al. (2020)	China	2017	27	-	4036	1501	Adults	-	-	GAD-2	-	Increase	<0.001
Zhao et al. (2020)	China	2017	27	-	4036	1501	Adults	-	-	PSS-4	-	Increase	<0.001
Psychiatric samples													
Liu et al. (2021)	China	10/2019	2	-	76	76	Adults	48.5	Substance use disorder (heroin)	HAM-A	Repeated-measures ANOVA	Increase	<0.01
Liu et al. (2021)	China	10/2019	6	-	76	76	Adults	48.5	Substance use disorder (heroin)	PSS	Repeated-measures ANOVA	Increase	<0.01
Lugo-Marín et al. (2021)	Spain (Catalonia)	-	-	-	35	35	Adults	32.8	Autism spectrum disorder	SCL-90-R Anxiety subscale	Wilcoxon signed-rank test	Decrease	-
Pan et al. (2021)	The Netherlands	2006	39	58	1181	1181	Adults	56	Depression, anxiety, or OCD	PSWQ	Mixed-effects models	Increase	range, 0.0007–0.35
Rutherford et al. (2021)	USA	-	1	-	46	46	Older adults	62.5	PTSD	HARS	-	-	-
Yocum et al. (2021)	USA	-	12	62	345	345	Adults	49	Bipolar diagnosis	GAD-7	Generalized estimating equations	Decrease	0.32
Child and adolescent psychiatric samples													
Breaux et al. (2021)	USA	09/2018	3	90.8	238	238	Children and adolescents	-	ADHD (approximately half of sample)	RCADS Anxiety subscale	Repeated-measures ANOVA	Increase	0.008
Conti et al. (2020)	Italy	-	7	-	61	61	Children and adolescents	-	Neurological and psychiatric disorders	CBCL 1.5–5	Paired *t* test	Increase	<0.05
Other selected samples													
Child and adolescent	samples												
Arjmand et al. (2021)	Australia	11/2/2018	3	-	775	775	Children and adolescents	-	-	PHQ-4	Multilevel modeling	Increase	< 0.05
De France et al. (2021)	Canada	-		73.9	184	136	Children and adolescents	14.12	-	MASC	Linear latent growth model	Increase	-
Luijten et al. (2021)	The Netherlands	12/2017	20	-	1318	813	Children and adolescents	13.2	-	PROMIS	Mean difference	Increase	<0.01
Magson et al. (2021)	Australia	2019	5	53	248	248	Children and adolescents	14.4	-	SCA-S Generalized Anxiety subscale	Paired *t* test	Increase	<0.001
Rogers et al. (2021)	USA	10/2019	6	66.8	407	407	Children and adolescents	15.42	-	GAD-7	Paired *t* test	Increase	<0.001
Medically vulnerable samples													
Cooley et al. (2021)	USA	-	-	-	133	133	Adults	50.3	HIV diagnosis	HADS Anxiety subscale	Mean difference	Increase	-
Mink van der Molen et al. (2021)	The Netherlands	10/2013	77	66	3239	1051	Adults	56	Breast cancer survivors	HADS	χ^2^ test	-	-
Sacre et al. (2021)	Australia	2018	12	96	450	450	Adults	66	Type 2 diabetes	GAD-7	Multilevel modeling	-	0.46
Stojanov et al. (2020)	Serbia	2017	27	-	64	64	Adults	54.1	Myasthenia gravis (MG)	HAM-A	Mann-Whitney test	Increase	ns
Thombs et al. (2020)	Canada, France, UK, USA	07/2019	4	37.1	435	435	Adults	56.9	Systemic sclerosis	PROMIS Anxiety 4a	Standardized mean difference	Increase	-
Wong et al. (2020)		04/03/2018	12	90.8	583	583	Older adults	70.9	2 or more chronic health conditions	GAD-7	Paired *t* test	Increase	0.011
Zambelli, Fidalgo, Halstead, and Dimitriou (2021)		02/2020	1	52	636	636	Adults	42.9	Chronic pain	HADS Anxiety subscale	Paired *t* test	-	0.713
Older adult samples													
Herrera et al. (2021)	Chile	11/2019	5	-	721	721	Older adults	71.59	-	GAI-SF	Paired *t* test	Increase	<0.001
Krendl and Perry (2021)	USA	06/2019	7	78.3	120	94	Older adults	74.9	-	GAD-7	-	Increase	-
Rutherford et al. (2021)	USA	-	1	-	30	30	Older adults	67.4	Trauma exposed comparison group	HARS	-	-	-
Siew et al. (2021)	Singapore	-	5	-	411	411	Older adults	69	-	GAI	Pearson’s correlation	Increase	ns
Other selected samples													
Flentje et al. (2020)	USA	06/2019	9	-	2288	2288	Adults	36.9	Sexual and gender minorities	GAD-7	Paired *t* test	Increase	<0.001
Loret de Mola et al. (2021)	Brazil	1/1/2019	5	-	1028	1028	Adults	27.5	Pregnant women	GAD-7	Mixed-effects models	Increase	<0.001
Hill et al. (2021)	USA	11/2019	7	75.6	3078	3078	Older adults	63.2	Veterans	PHQ-4	McNemar’s test	Increase	<0.001
Depression													
Unselected and undergraduate samples													
Algattas et al. (2021)	USA	1/1/2020	1	89.5	19	17	Adults	-	Neurosurgery residents	IDS-30	Paired *t* test	Decrease	0.397
Ayuso-Mateos et al. (2021)	Spain	6/17/2019	3	57	1103	1103	Adults	54.82	-	CIDI-Depression	McNemar’s test	Decrease	0.216
Brunoni et al. (2021)	Brazil	2008	14	51.7	2117	-	Adults	62.32	-	CIS-R	Cochran’s Q test for paired data	Decrease	ns
Bussone et al. (2020)	USA	-	6	-	68	68	Adults	-	Undergraduate students	SCL-90-R Depression subscale	Repeated-measures ANOVA	Increase	0.004
Campos et al. (2021)		08/2019	6	22.4	294	66	Adults	21.7	Pharmacy students	DASS-21 Depression subscale	Prevalence rates	Increase	-
Cooley et al. (2021)	USA	-	-	-	54	54	Adults	48.5	-	BDI-II	Mean difference	Increase	-
Ebrahimi, Hoffart, and Johnson (2021)		2015	50	-	1944	10 061	Adults	36	-	PHQ-9	Prevalence rates	-	-
Elmer et al. (2020)	Switzerland	2018	7	-	212	212	Adults	-	Undergraduate students	CES-D	Paired *t* test	Increase	<0.001
Emery et al. (2021)	USA	2017	16	46	720	670	Adults	25.19	-	6 item scale (Kandel and Davies, 1982)	-	-	-
Ergenekon et al. (2021)	Turkey	2017	36	-	21	21	Adults	40.6	Mothers of children on home ventilation	BDI	-	-	0.09
Evans et al. (2021)	UK	10/2019	5	84.1	259	259	Adults	19.76	Undergraduate students	HADS	Repeated-measures ANOVA	Increase	<0.001
Feinberg et al. (2021)	USA	2017	3	53.6	206	206	Adults	-	Parents	CES-D	Hierarchical linear modeling	Increase	<0.001
Gosselin et al. (2021)	France	-	3	-	100	100	Adults	-	-	PHQ-9	Paired McNemar χ^2^ test	Increase	0.17
Haliwa et al. (2021)		09/2019	3	36	146	146	Adults	43.75	MTurk sample (2 of 3)	DASS-21 Depression subscale	Paired *t* test	-	0.92
Haliwa et al. (2021)		09/2019	3	58	300	300	Adults	41.38	MTurk sample (1 of 3)	PHQ-8	Paired *t* test	-	0.585
Haliwa et al. (2021)		09/2019	3	35	142	142	Adults	40.46	MTurk sample (3 of 3)	PHQ-8	Paired *t* test	-	0.62
Hamadani et al. (2020)	Bangladesh	07/2017	15	72.8	2424	2424	Adults	24.1	-	CES-D	Interrupted time series analysis	Increase	<0.001
Hyland et al. (2021)	Ireland	02/2019	13	-	1020	1041	Adults	44.04	-	PHQ-9	Structural equation modeling	Decrease	<0.001
Ilgen et al. (2021)	Poland	-	-	-	99	99	Adults	35	-	BDI	Paired *t* test	Increase	<0.01
Lau et al. (2021)	England	-	3	-	104	104	Adults	-	Undergraduate students	PHQ-9	Paired *t* test	Increase	ns
Lee et al. (2020)	USA	01/2020	3	95	564	564	Adults	25.1	-	PHQ-4	Count ratios	Increase	0.013
Minhas et al. (2021)	Canada	-	-	73	473	473	Adults	23.8	-	PHQ-9	Linear mixed-effects models	Increase	<0.001
Novotný et al. (2020)	Czech Republic	-	-	39.2	715	715	Adults	46.12	-	PHQ	Wilcoxon signed-rank test	Increase	<0.001
Pan et al. (2021)	The Netherlands	2006	39	58	336	336	Adults	57.7	Healthy controls	QIDS	Mixed-effects models	Increase	<0.0001
Peters et al. (2020)	Germany	-	1	55.5	113 928	113 928	Adults	50	-	PHQ-9	-	Increase	-
Ramiz et al. (2021)	France	2014	3.5	12.8	1237	1237	Adults	62	-	PHQ-9	-	-	-
Saraswathi et al. (2020)	India	12/2019	6	90.8	217	217	Adults	20	Undergraduate students	DASS-21 Depression subscale	Wilcoxon signed-rank test	Increase	0.146
Winkler et al. (2021)	Czech Republic	2017	30	-	3306	3021	Adults	47.87	-	MINI	-	-	-
Yang, Ji, et al. (2021)	China	10/2019	4	-	2364	2364	Adults	20.04	Undergraduate students	DASS-21 Depression subscale	Paired *t* test	Decrease	<0.001
Yang, Hu, et al. (2021)	China	-	6	-	195	195	Adults	-	Undergraduate students	CES-D	Repeated-measures ANOVA	Increase	<0.001
Yocum et al. (2021)	USA	-	12	62	147	147	Adults	49	-	PHQ-9	Generalized estimating equations	Decrease	<0.0001
Zhang et al. (2020)		01/2020	-	100	49	49	Adults	-	Undergraduate students	PHQ-9	-	-	-
Zhao et al. (2020)	China	2017	27	-	4036	1501	Adults	-	-	PHQ-2	-	Increase	<0.001
Psychiatric samples													
Adams et al. (2021)	USA	3/11/2020	2	87.3	275	275	Adults	26.45	Autism spectrum disorder	DASS-21 Depression subscale	Repeated-measures ANCOVA	-	ns
Giel et al. (2021)	Germany	-	-	52	42	42	Adults	43.4	Eating disorder with frequent binge eating episodes	BDI	Wald χ^2^	Increase	0.02
Liu et al. (2021)	China	10/2019	2	-	76	76	Adults	48.5	Substance use disorder (heroin)	HAM-D	Repeated-measures ANOVA	Increase	<0.01
Lugo-Marín et al. (2021)	Spain (Catalonia)	-	-	-	35	35	Adults	32.8	Autism spectrum disorder	SCL-90-R Depression subscale	Wilcoxon signed-rank test	Decrease	-
Pan et al. (2021)	The Netherlands	2006	39	58	1181	1181	Adults	56	Depression, anxiety, or OCD	QIDS	Mixed-effects models	Increase	range, 0.0038–0.73
Yocum et al. (2021)	USA	-	12	62	345	345	Adults	49	Bipolar diagnosis	PHQ-9	Generalized estimating equations	Decrease	0.15
Child and adolescent psychiatric samples													
Breaux et al. (2021)	USA	09/2018	3	90.8	238	238	Children and adolescents	-	ADHD (approximately half of sample)	RCADS Depression subscale	Repeated-measures ANOVA	Increase	<0.001
Soriano-Ferrer et al. (2021)	Spain	11/2019	3	-	32	32	Children and adolescents	10.96	Dyslexia	CDI-S	Paired *t* test	Increase	0.001
Other selected samples													
Child and adolescent	samples												
Arjmand et al. (2021)	Australia	11/2/2018	3	-	775	775	Children and adolescents	-	-	PHQ-4	Multilevel modeling	Increase	<0.01
De France et al. (2021)	Canada	-	-	73.9	184	136	Children and adolescents	14.12	-	CDI	Linear latent growth model	Increase	-
Luijten et al. (2021)	The Netherlands	12/2017	20	-	1318	813	Children and adolescents	13.2	-	PROMIS	Mean difference	Increase	<0.01
Magson et al. (2021)	Australia	2019	5	53	248	248	Children and adolescents	14.4	-	SMFQ-C	Paired *t* test	Increase	<0.001
Rogers et al. (2021)	USA	10/2019	6	66.8	407	407	Children and adolescents	15.42	-	CDI-S	Paired *t* test	Increase	<0.001
Teng et al. (2021)	China	10/2019	5	95.5	1778	1778	Children and adolescents	-	Videogame players	CES-D	Paired *t* test	-	0.09
Thorisdottir et al. (2021)	Iceland	02/2018	12	-	3665	3123	Children and adolescents	15	-	SCL-90 Depression dimension	Standardized mean difference	Increase	-
Thorisdottir et al. (2021)	Iceland	02/2018	12	-	3494	3013	Children and adolescents	16	-	SCL-90 Depression dimension	Standardized mean difference	Increase	-
Thorisdottir et al. (2021)	Iceland	02/2018	12	-	3846	3421	Children and adolescents	14	-	SCL-90 Depression dimension	Standardized mean difference	Increase	-
Thorisdottir et al. (2021)	Iceland	10/2018	12	-	3900	3292	Children and adolescents	13	-	SCL-90 Depression dimension	Standardized mean difference	Increase	-
Thorisdottir et al. (2021)	Iceland	10/2018	12	-	2819	2080	Children and adolescents	18	-	SCL-90 Depression dimension	Standardized mean difference	Increase	-
Thorisdottir et al. (2021)	Iceland	10/2018	12	-	3098	2546	Children and adolescents	17	-	SCL-90 Depression dimension	Standardized mean difference	Increase	-
Medically vulnerable samples													
Capuano et al. (2021)	Italy	09/2019	4	-	75	67	Adults	37.5	Multiple sclerosis	BDI-II	Paired *t* test	-	0.117
Cooley et al. (2021)	USA	-	-	-	133	133	Adults	50.3	HIV diagnosis	BDI-II	Mean difference	Increase	-
Gul (2021)	Turkey	10/2019	6	-	116	116	Adults	-	Epilepsy	BDI	-	Increase	0.048
Mink van der Molen et al. (2021)	The Netherlands	10/2013	77	66	3239	1051	Adults	56	Breast cancer survivors	HADS	χ^2^ test	-	-
Sacre et al. (2021)	Australia	2018	12	96	450	450	Adults	66	Type 2 diabetes	PHQ-8	Multilevel modeling	-	0.98
Stojanov et al. (2020)	Serbia	2017	27	-	64	64	Adults	54.1	Myasthenia gravis (MG)	HAMD	Mann-Whitney test	Increase	ns
Strizović et al. (2021)	Serbia	12/15/2019	11	82.2	97	97	Adults	36	Epilepsy	NVDDI-E, Serbian version	Paired t test	Increase	<0.001
Thombs et al. (2020)	Canada, France, UK, USA	07/2019	4	37.1	388	388	Adults	56.9	Systemic sclerosis	PHQ-8	Standardized mean difference	Decrease	-
Ugurlucan et al. (2021)		01/2018	6	67.5	77	77	Adults	30.2	Vaginismus	BDI	Paired *t* test	Increase	0.02
Villani et al. (2020)		-	1	20.8	46	46	Adults	40.6	Down syndrome	DRS	Sign test for matched data	Increase	0.032
Yildirim et al. (2021)	Turkey	02/03/2020	1	93.4	595	595	Adults	50.58	Breast, ovarian, colorectal, or gastric cancer	BDI	-	Increase	-
Zambelli et al. (2021)		02/2020	1	52	636	636	Adults	42.9	Chronic pain	HADS-D	Paired *t* test	Decrease	0.001
Older adult samples													
Barcellos et al. (2021)	USA	11/2019	3	64	16 644	16 644	Older adults	64.3	-	PHQ-2	Paired test of mean differences	Increase	<0.001
Hamm et al. (2020)	USA	-	1	66.4	73	73	Older adults	69.2	-	PHQ-9	Paired *t* test	Decrease	0.8
Herrera et al. (2021)	Chile	11/2019	5	-	720	720	Older adults	71.59	-	PHQ-9	Paired *t* test	Increase	<0.001
Krendl and Perry (2021)	USA	06/2019	7	78.3	120	94	Older adults	74.9	-	PHQ-4	Paired *t* test	Increase	0.003
McArthur et al. (2021)	USA	01/2017	-	-	765	-	Older adults	81.4	-	interRAI LTCF - DRS	χ^2^ test	Decrease	<0.002
Mishra et al. (2021)	USA	-	6	-	10	10	Older adults	77.3	Elevated risk of falling	CES-D	Paired *t* test	Increase	0.046
Nogueira et al. (2021)	Portugal	-	17	59.5	150	150	Older adults	69	-	GSD-30	Paired *t* test	Increase	0.001
Rutherford et al. (2021)	USA	-	1	-	30	30	Older adults	67.4	Trauma exposed comparisons	HRSD	Generalized linear mixed models	Increase	0.025
Rutherford et al. (2021)	USA	-	1	-	46	46	Older adults	62.5	PTSD	HRSD	Generalized linear mixed models	Increase	0.181
Wong et al. (2020)	Hong Kong	04/03/2018	12	90.8	583	583	Older adults	70.9	2 or more chronic health conditions	PHQ-9	Paired *t* test	Increase	0.359
Other selected samples													
Flentje et al. (2020)	USA	06/2019	9	-	2288	2288	Adults	36.9	Sexual and gender minorities	PHQ-9	Paired *t* test	Increase	<0.001
Fruehwirth et al. (2021)	USA	1/2020	4	42	419	419	Adults	18.9	Sexual and gender minority students	PHQ-8	Prevalence rates	Increase	<0.001
Hill et al. (2021)	USA		7	75.6	4069	3078	Older adults	63.2	Veterans	PHQ-4	McNemar’s test	-	0.07
King, Feddoes, Kirshenbaum, Humphreys, and Gotlib (2021)	USA	02/2017	11	-	82	82	Adults	33.56	Pregnant women	EPDS	Welch’s *t* test	Increase	<0.001
Lorentz et al. (2021)	Brazil	11/2019	-	40	50	50	Adults	25	Pregnant women	EPDS	Friedman’s test	Increase	0.004
Loret de Mola et al. (2021)	Brazil	1/1/2019	5	-	1042	1042	Adults	27.5	Pregnant women	EPDS	Mixed-effects models	Increase	<0.001
Perzow et al. (2021)	USA	- (average 3.8 months prepandemic)		-	135	135	Adults	31.81	Pregnant women oversampled for elevated depression symptoms	EPDS	Repeated-measures ANOVA	Increase	<0.001
General distress													
Unselected and undergraduate samples													
Achterberg et al. (2021)	The Netherlands	2019	-	52	99	105	Adults	44.89	-	BSI	Friedman’s test	Increase	0.002
Bussone et al. (2020)	USA	-	6	-	68	68	Adults	-	Undergraduate students	STAI-Y	Repeated-measures ANOVA	Increase	<0.001
Chandola et al. (2020)	UK	2019	1	49	-	13 754	Adults	-	-	GHQ-12	Prevalence rates	-	-
Ergenekon et al. (2021)	Turkey	2017	36	-	21	21	Adults	40.6	Mothers of children on home ventilation	STAI-T	-	-	0.46
da Freitas et al. (2021)	Brazil	03/2019	7	52.2	71	71	Adults	21.26	Undergraduate students	STAI	Paired *t* test	Decrease	0.047
Gagné et al. (2021)	Canada	03/2019	12	77	127	127	Adults	-	-	K10	ANOVA	Increase	0.046
Li et al. (2020)	China	12/20/2019	2	88.9	555	555	Adults	19.6	Undergraduate students	PHQ-4	Paired *t* test	Decrease	<0.001
Seitz et al. (2021)	Germany	09/2018	5	60.7	22	22	Adults	31.3	Healthy volunteers	BSI	Paired *t* test	Increase	-
Soriano-Ferrer et al. (2021)	Spain	11/2019	3	-	32	32	Adults	42.2	Mothers of children with dyslexia	PSI-SF	Paired *t* test	Increase	0.001
Twenge and Joiner (2020)	USA	2018	16	-	19 330	2032	Adults	44.7	-	K6	Mean difference	Increase	-
van Zyl, Rothmann, and Zondervan-Zwijnenburg (2021)	The Netherlands	01/2020			141	141	Adults			Mental Health Continuum Short Form	Latent growth model		ns
Psychiatric samples													
Lugo-Marín et al. (2021)	Spain (Catalonia)	-	-	-	35	35	Adults	32.8	Autism spectrum disorder	SCL-90-R	Wilcoxon signed-rank test	Decrease	-
Lugo-Marín et al. (2021)	Spain (Catalonia)	-	-	-	37	37	Children and adolescents	10.7	Autism spectrum disorder	CBCL Anxious/Depressed subscale	Wilcoxon signed-rank test	-	-
Seitz et al. (2021)	Germany	09/2018	5	60.7	63	63	Adults	31.3	MDD, PTSD, or somatic symptom disorder	BSI	Paired *t* test	Increase	-
Soriano-Ferrer et al. (2021)	Spain	11/2019	3	-	32	32	Children and adolescents	10.96	Dyslexia	STAIC	Paired *t* test	Increase	0.001
Other selected samples													
Child and adolescent samples													
Achterberg et al. (2021)	The Netherlands	2019	-	37	203	209	Children and adolescents	12	-	SDQ	Friedman’s test	Increase	-
Gagné et al. (2021)	Canada	03/2019	12	77	127	127	Children and adolescents	10	-	SDQ	ANOVA	Decrease	<0.001
Teng et al. (2021)	China	10/2019	5	95.5	1778	1778	Children and adolescents	-	Videogame players	STAI	Paired t test	Increase	0.004
Medically vulnerable samples													
Asquini, Bianchi, Borromeo, Locatelli, and Falla (2021)	Italy	7/2019	4	89	40	40	Adults	-	Temporomandibular disorders (TMDs)	HADS	Median difference	Increase	-
Ayaz et al. (2020)	Turkey	-	-	-	63	63	Adults	30.35	Pregnant women	IDAS-II	-	Increase	<0.001
Bonenkamp et al. (2021)	The Netherlands	12/2017	3	-	177	177	Adults	64.9	Dialysis patients	Mental Component Summary (MCS) score of 12-item Short Form (SF-12) health survey	Wilcoxon signed-rank test	Increase	0.2
Capuano et al. (2021)	Italy	09/2019	4	-	75	67	Adults	37.5	Multiple sclerosis	STAI-T	Paired *t* test	-	0.319
Kidd et al. (2021)	USA	2019	3	76.4	208	208	Adults	37.8	Transgender and gender non-binary individuals	BSI-18	Paired *t* test	Increase	0.008
Perzow et al. (2021)	USA	-	-	-	135	135	Adults	31.81	Pregnant women oversampled for elevated depression symptoms	STAI-SF	Repeated-measures ANOVA	Increase	<0.001
Rivera et al. (2021)	Spain	-	-	54.8	51	51	Adults	48.2	Fibromyalgia	Combined Index of Severity in Fibromyalgia	Repeated-measures ANOVA	Decrease	0.604

OCD, obsessive-compulsive disorder; MDD, major depressive disorder; HIV, human immunodeficiency virus; Y-BOCS, Yale-Brown Obsessive Compulsive Scale; CIS-R, Clinical Interview Schedule-Revised; CBCL, Child Behaviour Check List; OCI-R, Obsessive-Compulsive Inventory-Revised; PCL-5, PTSD Checklist for DSM-5; SCL-90-R, Symptom Checklist 90 Revised; CY-BOCS, Children’s Yale-Brown Obsessive Compulsive Scale; DASS, Depression Anxiety Stress Scale (42 or 21 item version specified); BAI, Beck Anxiety Subscale; PHQ, Patient Health Questionnaire (2, 4, 8, or 9-item version specified); PSWQ, Penn State Worry Questionnaire; RCADS, Revised Child Anxiety and Depression Scales; HADS, Hospital Anxiety and Depression Scales; GAD, Generalized Anxiety Disorder (2 or 7-item version specified); MASC, Multidimensional Anxiety Inventory for Children; PSS, Perceived Stress Scale (4-item version specified); GAI, Geriatric Anxiety Inventory (Short Form [SF] specified); HAM-A, Hamilton Anxiety Scale; PROMIS, Patient-Reported Outcomes Measurement Information System; SCA-S, Spence Children’s Anxiety Scale; MINI, Mini International Neuropsychiatric Interview; IDS-30, Inventory of Depressive Symptomology 30; CIDI, Composite International Diagnostic Interview; CDI, Children’s Depression Inventory (Short Form [S] specified); CES-D, Center for Epidemiologic Studies Depression Scale; EPDS, Edinburgh Postnatal Depression Scale; HAM-D, Hamilton Depression Scale; SMFQ-C, Short Mood and Feelings Questionnaire – Child Version; GDS, Geriatric Depression Scale; QIDS, Quick Inventory of Depressive Symptoms; NVDDI-E, Neurological Disorders Depression Inventory for Epilepsy; DRS, Depression Rating Scale; BSI, Brief Symptom Inventory; SDQ, Strengths and Difficulties Questionnaire; IDAS-II, Inventory of Depression and Anxiety Symptoms – II; STAI, State-Trait Anxiety Inventory; GHQ-12, General Health Questionnaire; K10 or K6, Kessler Psychological Distress Scale (10 or 6-item version); PSI-SF, Parenting Stress Index – Short Form.

*Note*. In many cases, authors only reported T1 data (e.g. sample size) for participants for whom T2 data were also available. An equivalent *N* at T1 and T2 may be indicative of participants with available data at both timepoints, rather than the total sample being the same size at both timpoints. Response rate is reported as the percentage of participants with available data at T1 who responded to an invitation to participate in T2 follow-up data collection. Many studies invited only a subset of participants from T1 to participate at T2; hence, response rate is not always obtained by diving *N* at T2 by *N* at T1.
