# Supplementary material for: A narrative systematic review of changes in mental health symptoms from before to during the COVID-19 pandemic
Source: Psychol Med. Author manuscript; Available in PMC 2025 Jan 7. (PMC11706599; doi:10.1017/S0033291723002295)
Supplement: supplemental materials [file NIHMS2043633-supplement-supplemental_materials.docx]

**COVID-19 Mental Health Workgroup**

*Member list and project-level CRediT author statement*

Gordon J. G. Asmundson – conceptualization (supporting)

Rosanna Breaux – conceptualization (lead), methodology (major), resources (major), project administration (lead)

Katie Burkhouse – conceptualization (supporting), resources (major)

M. Kathleen Caulfield – data curation (lead), formal analysis (supporting), investigation (major)

Christine Cha – conceptualization (major)

Samuel Cooper – conceptualization (major)

Darlene Davis-Goodwine – conceptualization (supporting), methodology (supporting)

Edwin Dalmaijer – data curation (major)

Eiko I. Fried – conceptualization (supporting)

Ilana Gratch – conceptualization (supporting)

Lauren S. Hallion – conceptualization (lead), methodology (major), resources (lead), project administration (lead)

Benjamin A. Katz – conceptualization (major), methodology (major), resources (major), project administration (major)

Philip Kendall – conceptualization (supporting)

Susan Kusmierski – investigation (lead)

Katie Kriegshauser – conceptualization (supporting), resources (major)

Cecile Ladouceur – conceptualization (supporting), methodology (major)

Shmuel Lissek – resources (major)

Adrienne Manbeck – conceptualization (supporting), investigation (supporting)

Tyler McFayden – conceptualization (supporting), investigation (supporting)

Kristin Naragon-Gainey – conceptualization (major), methodology (major)

Rebecca Price – resources (major)

Kathryn A. Roecklein – resources (major)

Lisa R. Starr – conceptualization (major), methodology (major), resources (major), project administration (supporting)

Jeremy G. Stewart – conceptualization (major), methodology (major), resources (major), project administration (supporting)

Bethany A. Teachman – conceptualization (major), methodology (major), resources (lead), project administration (major)

Aidan G. C. Wright – resources (major)

Iftah Yovel – resources (supporting)

| **Table S1** |  |  |
| --- | --- | --- |
| *Demographic and clinical characteristics (N = 198)* |  |  |
|  | *n* | *%* |
| Gender |  |  |
| Man | 34 | 17 |
| Woman | 160 | 81 |
| Non-binary | 2 | 1 |
| Transgender man | 2 | 1 |
| Age *(M, SD)* | 37.94 | 15 |
| Race |  |  |
| American Indian or Alaska Native | 3 | 2 |
| Asian | 18 | 19 |
| Black or African-American | 11 | 6 |
| White | 171 | 86 |
| Other race | 5 | 3 |
| Ethnicity |  |  |
| Hispanic or Latinx | 10 | 5 |
| Non-Hispanic or Latinx | 188 | 95 |
| Nationality |  |  |
| United States of America | 190 | 96 |
| United Kingdom | 2 | 1 |
| Canada | 3 | 2 |
| European (Spain, Poland, Belgium) | 3 | 2 |
|  |  |  |
| Level of education |  |  |
| Four-year college or bachelor’s degree | 74 | 37 |
| Master’s degree or equivalent | 54 | 27 |
| Some college or associate’s degree (American system) | 35 | 18 |
| Doctoral degree or equivalent | 18 | 9 |
| High school or GED (American system) | 14 | 7 |
| A-levels (British system) | 1 | 1 |
| GCSE (British system) | 1 | 1 |
| Primary education only | 1 | 1 |
| Sexual orientation |  |  |
| Straight/heterosexual | 153 | 77 |
| Bisexual | 27 | 14 |
| Gay/homosexual | 9 | 5 |
| Other sexual orientation | 9 | 5 |
| *Note.* Gender response options were mutually exclusive; e.g., participants were able to select only one. “Transgender woman” was included as an option but no participants in the included sample selected this option. | | |

| **Table S2** | | | | | |  |
| --- | --- | --- | --- | --- | --- | --- |
| *Within-person associations between anxiety, worry, and difficulty concentrating* | | | | | |  |
| Variables | 1 | 2 | 3 | 4 | 5 | |
| 1. DASS-21_Anx_ |  |  |  |  |  | |
| 2. PSWQ-PW | 0.37*** |  |  |  |  | |
| 3. ACS | 0.17*** | 0.28*** |  |  |  | |
| 4. DASS-21_Dep_ | 0.43*** | 0.27*** | 0.20*** |  |  | |
| 5. PROMIS Sleep-SF | 0.15** | 0.16** | 0.13** | 0.02 |  | |
| 6. CRISIS Worries | 0.21*** | 0.26*** | 0.17** | 0.15** | 0.16** | |
| *Note.* DASS-21_Anx_, Depression Anxiety Stress Scales 21 Anxiety composite; PSWQ-PW, Penn State Worry Questionnaire – Past Week; ACS, Attentional Control Scale; DASS-21_Dep_, Depression Anxiety Stress Scales 21 Depression subscale; PROMIS Sleep-SF, PATIENT-REPORTED OUTCOMES MEASUREMENT INFORMATION SYSTEM Sleep Disturbance - Short Form; CRISIS Worries, Coronavirus Health Impact Survey COVID Worries Domain. Degrees of freedom for repeated-measures correlations ranged from 364-394.  * *p* < .05, ***p* < .01, ****p* < .001. | | | | | |  |

**Competing mediation model.** To probe the specificity of the hypothesized mediation model, we also tested a competing path model in which anxiety at T2 was evaluated as a mediator of the association between worry at T1 and difficulty concentrating at T3. The results of this competing path model were consistent with partial mediation (total effect of worry at T1 on difficulty concentrating at T3: *b* = 0.15, *SE* = 0.03, *β* = 0.33, CI = [0.08 – 0.21]; indirect effect of worry at T1 on difficulty concentrating at T3 through anxiety at T2: *b* = 0.11, *SE* = 0.02, *β* = 0.24, CI = [0.07 – 0.15]; direct effect of worry at T1 on difficulty concentrating at T3 (*b* = 0.04, *SE* = 0.04, *β* = 0.10, CI = [-0.03 – 0.12]). See Figure 4 for other path coefficients. Effect size was evaluated as the ratio of the indirect effect to the total effect (*b* = 0.71, *SE* = 0.24, *β* = 0.71, CI = [0.40 – 1.30]). This can be interpreted to mean that 71% of the total effect of worry at T1 on difficulty concentrating at T3 is statistically attributable to anxiety at T2 in the specified model. This competing model was rerun with covariates in the same manner described above for the original mediation model; see Figures 5 and 6 for path coefficients.

**Individual regression paths.** We ran separate models regressing each outcome on its predictor at the previous timepoint, including that outcome at the previous timepoint as a covariate (Table 8). Anxiety at T1 was significantly associated with worry at T2, controlling for worry at T1 (*b* = 0.26, *SE* = 0.12, *β* = 0.10, *p* = .039). In all other models, only the outcome at the previous timepoint was significantly associated with the outcome.

**Patterns of missingness.** The original dataset provided to the lead author contained data from 1203 participants, all of whom had provided data for at least one assessment wave, but a majority of whom had not responded to one or more of the three assessment waves in the dataset. Patterns of missingness in this original dataset were evaluated using Little’s Missing Completely at Random (MCAR) test. Little’s test statistic was significant (*l*(*df*=339) = 406.55, *p* = .007), suggesting non-ignorable patterns of missingness in the data. Of the 1203 participants who provided data at any assessment wave, 372 participants had complete data at T1 and T3, but were missing data on all measures at T2. An additional 336 participants had complete data at T1, but were missing data on all measures at T2 and T3. Chi-square tests indicated that individuals missing data at T2 and T3 were significantly more likely to have only a high school degree or GED, be full-time students, or be unemployed due to student status.

To further investigate the possibility of data missing not at random (MNAR) in the original dataset (*N* = 1203), we conducted a series of logistic regression analyses. Each of the primary analytic variables (PSWQ, ACS, DASS-21 Stress and Anxiety subscales) at Wave *t* was entered as a predictor of missing data on any primary analytic variables at Wave *t+1* (e.g., Wave 1 to Wave 2; Wave 2 to Wave 3). None of the primary analytic variables emerged as a significant predictor of missingness at the next wave in any logistic regression model, suggesting that participants’ scores on measures of worry, anxiety, and difficulty concentrating were not associated with the likelihood that they would fail to provide data at the next wave.

The participants included in analyses (*N* = 198) provided data on at least one primary analytic variable (DASS-21 Anxiety composite, PSWQ, ACS) at all three timepoints. Of 594 observations obtained from 198 participants across all three timepoints, 25 observations (4.20%) were missing PSWQ data only, 14 observations (2.36%) were missing ACS data only, 8 observations (1.35%) were missing both PSWQ and ACS data, and 3 observations (0.51%) were missing DASS-21 Anxiety data only. Overall, 50 observations (8.42%) from included participants were missing data on at least one primary analytic variable.

Patterns of missingness in the included sample of 198 participants were evaluated using Little’s Missing Completely at Random (MCAR) test. Little’s test statistic was significant (*l*(*df*=20) = 33.05, *p* = .033), suggesting non-ignorable patterns of missingness in the data. Chi-square tests indicated that individuals missing data on at least one primary analytic variable were significantly more likely to identify as gay or bisexual than individuals with no missing data.

**Multicollinearity.** We computed variance inflation factors (VIFs) for the models used to test each of the three main hypotheses. As there is no field-standard approach to computing VIFs for multilevel models, two approaches were compared. First, the multilevel models for H1 and H2 were converted to simple linear regressions (fixed effects only), and VIFs were computed for each model. Second, a specialized VIF that accounts for multilevel structure was computed for the multilevel models testing H1 and H2. VIFs were close to identical between the two methods (e.g., differences of no more than 0.01). The main predictors (e.g., fixed effects) in the models for H1 and H2 had VIFs of 1 – 1.36, which is considerably below published VIF thresholds of 5, which indicates the presence of multicollinearity, or 10, which is indicates severe multicollinearity and potentially cause for concern (James et al., 2017). Variance inflation factors for the predictors in the structural equation models for H3 ranged from 1.09 to 4.14, but still did not exceed the minimum cutoff of 5 which would indicate the presence of multicollinearity.

**Table S3**

*Zero-order correlations at Wave 1*

|  | 1 | 2 | 3 | 4 | 5 |
| --- | --- | --- | --- | --- | --- |
| 1. DASS-21_Anx_ |  |  |  |  |  |
| 2. PSWQ | 0.69 |  |  |  |  |
| 3. ACS | 0.44 | 0.48 |  |  |  |
| 4. DASS-21_Dep_ | 0.66 | 0.54 | 0.46 |  |  |
| 5. PROMIS Sleep-SF | 0.34 | 0.26 | 0.18 | 0.27 |  |
| 6. CRISIS Worries | 0.49 | 0.48 | 0.27 | 0.40 | 0.29 |
| *Note.* DASS-21_Anx_, Depression Anxiety Stress Scales 21 Anxiety composite; PSWQ, Penn State Worry Questionnaire; ACS, Attentional Control Scale; DASS-21_Dep_, Depression Anxiety Stress Scales 21 Depression subscale; PROMIS Sleep-SF, Patient-Reported Outcomes Measurement Information System Sleep Disturbance - Short Form; CRISIS Worries, Coronavirus Health Impact Survey COVID Worries Domain. | | | | | |

**Table S4**

*Zero-order correlations at Wave 2*

|  | 1 | 2 | 3 | 4 | 5 | |
| --- | --- | --- | --- | --- | --- | --- |
| 1. DASS-21_Anx_ |  |  |  |  |  | |
| 2. PSWQ | 0.66 |  |  |  |  | |
| 3. ACS | 0.46 | 0.38 |  |  |  | |
| 4. DASS-21_Dep_ | 0.64 | 0.53 | 0.38 |  |  | |
| 5. PROMIS Sleep-SF | 0.30 | 0.22 | 0.11 | 0.16 |  | |
| 6. CRISIS Worries | 0.44 | 0.42 | 0.26 | 0.41 | 0.21 | |
| *Note.* DASS-21_Anx_, Depression Anxiety Stress Scales 21 Anxiety composite; PSWQ, Penn State Worry Questionnaire; ACS, Attentional Control Scale; DASS-21_Dep_, Depression Anxiety Stress Scales 21 Depression subscale; PROMIS Sleep-SF, Patient-Reported Outcomes Measurement Information System Sleep Disturbance - Short Form; CRISIS Worries, Coronavirus Health Impact Survey COVID Worries Domain. | | | | | |  |

**Table S5**

*Zero-order correlations at Wave 3*

|  | 1 | 2 | 3 | 4 | 5 |  |
| --- | --- | --- | --- | --- | --- | --- |
| 1. DASS-21_Anx_ |  |  |  |  |  |  |
| 2. PSWQ | 0.70 |  |  |  |  |  |
| 3. ACS | 0.51 | 0.54 |  |  |  |  |
| 4. DASS-21_Dep_ | 0.68 | 0.54 | 0.46 |  |  |  |
| 5. PROMIS Sleep-SF | 0.33 | 0.28 | 0.22 | 0.24 |  |  |
| 6. CRISIS Worries | 0.50 | 0.47 | 0.32 | 0.40 | 0.29 |  |
| *Note.* DASS-21_Anx_, Depression Anxiety Stress Scales 21 Anxiety composite; PSWQ, Penn State Worry Questionnaire; ACS, Attentional Control Scale; DASS-21_Dep_, Depression Anxiety Stress Scales 21 Depression subscale; PROMIS Sleep-SF, Patient-Reported Outcomes Measurement Information System Sleep Disturbance - Short Form; CRISIS Worries, Coronavirus Health Impact Survey COVID Worries Domain. | | | | | | |

| **Table S6**  *Exploratory regression paths from mediation analyses* | | | | | | |
| --- | --- | --- | --- | --- | --- | --- |
|  | *b* | *β* | *SE* | *t* | *p* |  |
| Model 1 (DV: PSWQ-PW at T2) |  |  |  |  |  |  |
| Intercept | 11.18 | -0.05 | 1.88 | 5.94 | < .001 |  |
| DASS-21_Anx_ at T1 | 0.26 | 0.10 | 0.12 | 2.08 | .039 |  |
| PSWQ-PW at T1 | 0.75 | 0.78 | 0.05 | 16.05 | < .001 |  |
| Model 2 (DV: PSWQ-PW at T3) |  |  |  |  |  |  |
| Intercept | 4.00 | 0.12 | 1.92 | 2.08 | .039 |  |
| DASS-21_Anx_ at T2 | -0.01 | -0.01 | 0.11 | -0.13 | .896 |  |
| PSWQ-PW at T2 | 0.92 | 0.836 | 0.05 | 19.88 | < .001 |  |
| Model 3 (DV: ACS at T2) |  |  |  |  |  |  |
| Intercept | 7.96 | -0.01 | 1.64 | 4.85 | < .001 |  |
| PSWQ-PW at T1 | 0.00 | 0.00 | 0.02 | 0.03 | .977 |  |
| ACS at T1 | 0.84 | 0.89 | 0.04 | 23.88 | < .001 |  |
| Model 4 (DV: ACS at T3) |  |  |  |  |  |  |
| Intercept | 5.72 | 0.17 | 1.98 | 2.89 | .004 |  |
| PSWQ-PW at T2 | 0.02 | 0.05 | 0.02 | 1.29 | .199 |  |
| ACS at T2 | 0.87 | 0.81 | 0.04 | 20.10 | < .001 |  |
| *Note.* DASS-21_Anx_, Depression Anxiety Stress Scales 21 Anxiety composite; PSWQ, Penn State Worry Questionnaire; ACS, Attentional Control Scale. | | | | | |  |

**Figure S1 – Competing mediation model.** Structural equation model with anxiety as a mediator of the association between worry and difficulty concentrating, without covariates (*N* = 185). Coefficients are standardized betas.


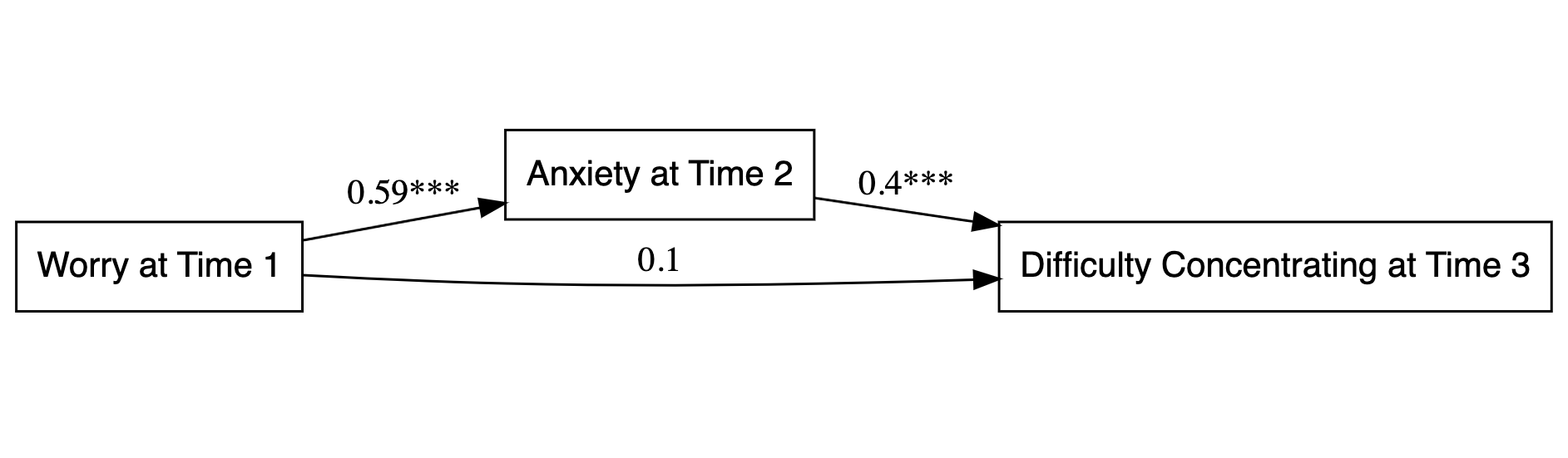


**Figure S2 – Competing mediation model with covariates.** Structural equation model with anxiety as a mediator of the association between worry and difficulty concentrating, regressing each outcome on itself at the previous timepoint (*N* = 180). Coefficients are standardized betas.


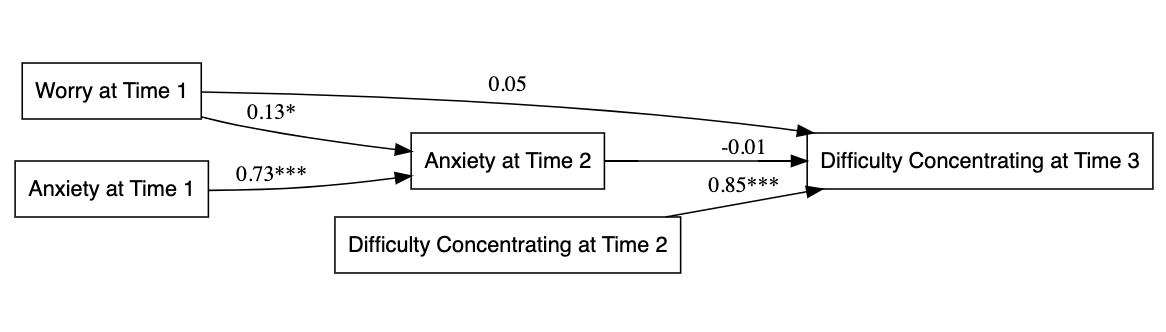


**Figure S3 – Competing mediation model with full covariates.** Structural equation model with anxiety as a mediator of the association between worry and difficulty concentrating, regressing each outcome on itself at the previous timepoint, and including depression, sleep disturbance, and difficulty concentrating at T1 as additional exogenous predictors of difficulty concentrating at T3 ( (*N* = 175). Coefficients are standardized betas.


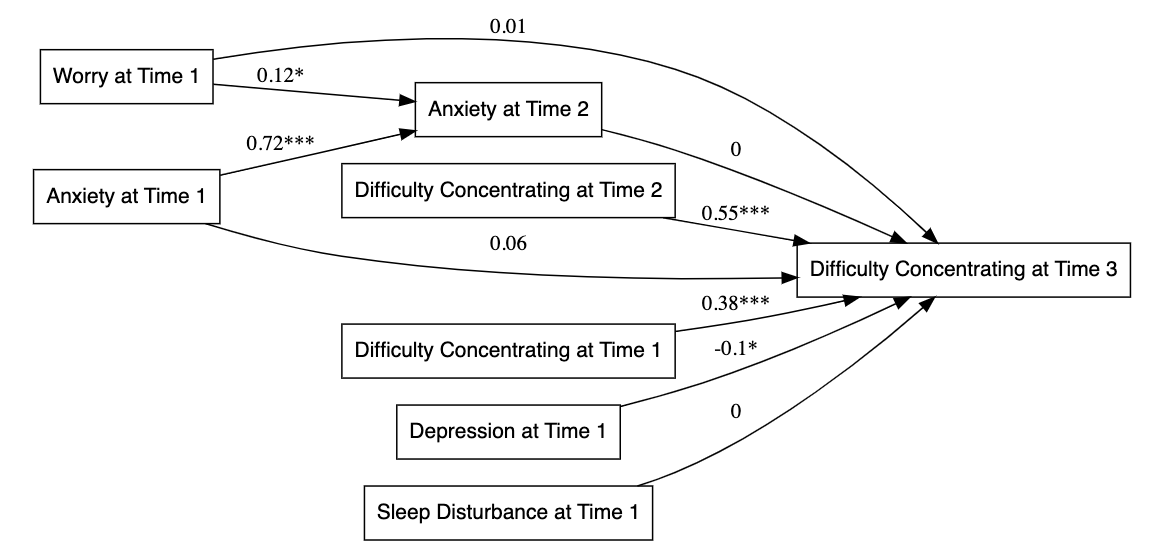


**Figure S4 – Original mediation model controlling for COVID worry at Time 1.** Structural equation model with anxiety as a mediator of the association between worry and difficulty concentrating, including COVID worry at Time 1 as an exogenous predictor of difficulty concentrating at Time 3 ( (*N* = 183). Coefficients are standardized betas.


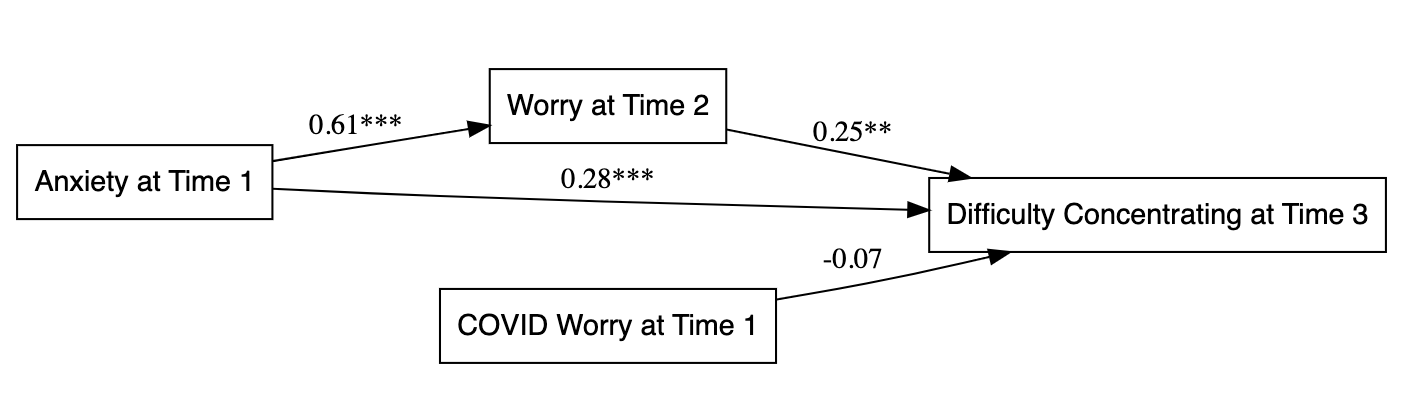


**RESULTS OF MODELS WITH ALL ITEMS INCLUDED.**

**Primary Analyses**

***Association Between Anxiety and Worry***

To test the first hypothesis, that anxiety would be significantly associated with worry within- and between-person, we constructed a multilevel model with timepoint, participant mean anxiety, and person-mean centered anxiety as fixed effects, participant as a random intercept, and worry as the dependent variable (Table 2). Anxiety was associated with worry both between (*b* = 1.81, *SE* = 0.13, *β* = 0.65, *p* < .001) and within (*b* = 0.84, *SE* = 0.11f, *β* = 0.12, *p* < .001) participants. This means that both an individual’s average level of anxiety (between-person) and deviations from their average level of anxiety at a given timepoint (within-person) were associated with their reported worry at the same given timepoint. Timepoint was positively associated with worry at T2 (*b* = 1.54, *SE* = 0.77, *β* = 0.07, *p* = .047), suggesting that worry was significantly higher at T2 compared to T1. When this model was rerun controlling for depression severity (Table 4), anxiety remained significantly associated with worry both between (*b* = 1.61, *SE* = 0.14, *β* = 0.58, *p* < .001) and within (*b* = 0.70, *SE* = 0.12, *β* = 0.02, *p* < .001) participants. Depression symptom severity was also significantly associated with worry (*b* = 0.22, *SE* = 0.11, *β* = 0.19, *p* = .003).

***Association Between Worry and Difficulty Concentrating***

To test the second hypothesis, that worry would be significantly associated with difficulty concentrating at the within- and between-person levels, we constructed a multilevel model with timepoint, participant mean worry, and person-mean centered worry as fixed effects, participant as a random intercept, and difficulty concentrating as the dependent variable (Table 3). Difficulty concentrating was associated worry both between (*b* = 0.19, *SE* = 0.03, *β* = 0.38, *p* < .001) and within (*b* = 0.12, *SE* = 0.02, *β* = 0.09, *p* < .001) participants. This means that both an individual’s average level of worry (between-person) and deviations from their average level of worry at a given timepoint (within-person) were associated with difficulty concentrating at the same given timepoint. Difficulty concentrating did not significantly vary between timepoints. When this model was rerun controlling for depression severity, sleep disturbance, and COVID-related worries (Table 4), worry remained significantly associated with difficulty concentrating both between (*b* = 0.14, *SE* = 0.03, *β* = 0.29, *p* < .001) and within (*b* = 0.10, *SE* = 0.02, *β* = 0.07, *p* < .001) participants. Depression symptom severity (*b* = 0.09, *SE* = 0.03, *β* = 0.10, *p* = .004) and sleep disturbance (*b* = 0.15, *SE* = 0.07, *β* = 0.03, *p* = .027) were significantly associated with difficulty concentrating, whereas COVID-related worries were not.

***Mediation Analysis***

To test the third hypothesis, that worry would fully mediate the relationship between anxiety and difficulty concentrating, we conducted a mediation analysis using structural equation modeling. All parameters were estimated simultaneously, standardized estimates were computed using all path information, and standard errors were computed through 5000 bootstrapped samples. The initial path model regressed difficulty concentrating at T3 on worry at T2 and anxiety at T1, and worry at T2 on anxiety at T1. All paths were significant (total effect of anxiety at T1 on difficulty concentrating at T3: *b* = 0.48, *SE* = 0.07, *β* = 0.42, CI = [0.34 – 0.62]; indirect effect of anxiety at T1 on difficulty concentrating at T3 through worry at T2: *b* = 0.16, *SE* = 0.06, *β* = 0.14, CI = [0.05 – 0.29]; direct effect of anxiety at T1 on difficulty concentrating at T3: *b* = 0.31, *SE* = 0.08, *β* = 0.28, CI = [0.15 – 0.48]). See Figure 1 for other path coefficients. Effect size was evaluated as the ratio of the indirect effect to the total effect (*b* = 0.34, *SE* = 0.13, *β* = 0.34, CI = [0.10 – 0.62]). This suggests that 34% of the total effect of anxiety on difficulty concentrating was statistically attributable to worry in the specified model, consistent with partial mediation. Our hypothesis of a full mediation was therefore rejected.

Post-hoc linear regression tests of the pairwise relationships between anxiety, worry, and difficulty concentrating from one timepoint to the next, controlling for each outcome at the previous timepoint, were conducted (see Table S6 in Supplemental Materials).

**Sensitivity Analyses**

The path model testing worry as a mediator of the association between anxiety and difficulty concentrating was rerun adding covariates in two steps. In the first step, regressing each outcome on itself at the previous timepoint, the main paths of interest were no longer significant (total effect of anxiety at T1 on difficulty concentrating at T3: *b* = 0.05, *SE* = 0.05, *β* = 0.04, CI = [-0.05 – 0.14]; indirect effect of anxiety at T1 on difficulty concentrating at T3 through worry at T2: *b* = 0.01, *SE* = 0.01, *β* = 0.01, CI = [-0.01 – 0.03]; direct effect of anxiety at T1 on difficulty concentrating at T3: *b* = 0.04, *SE* = 0.05, *β* = 0.04, CI = [-0.06 – 0.15]). See Figure 2 for other path coefficients. Effect size was evaluated as the ratio of the indirect effect to the total effect (*b* = 0.11, *SE* = 84.85, *β* = 0.11, CI = [-0.27 – 10.24]), suggesting that 11% of the total effect of anxiety at T1 on difficulty concentrating at T3 is statistically attributable to worry at T2 in the specified model.

In the second step, we added paths for worry, difficulty concentrating, depression symptom severity, and sleep disturbance at T1 and retained paths from the above model regressing each outcome on itself at the previous timepoint. With the addition of worry, difficulty concentrating, depression symptom severity, and sleep disturbance at T1 as exogenous predictors, the main paths of interest were no longer significant (total effect of anxiety at T1 on difficulty concentrating at T3: *b* = 0.07, *SE* = 0.07, *β* = 0.06, CI = [-0.07 – 0.20]; indirect effect of anxiety at T1 on difficulty concentrating at T3 through worry at T2: *b* = 0.01, *SE* = 0.07, *β* = 0.01, CI = [-0.01 – 0.05]; direct effect of anxiety at T1 on difficulty concentrating at T3: *b* = 0.05, *SE* = 0.07, *β* = 0.05, CI = [-0.09 – 0.19]). See Figure 3 for other path coefficients. Effect size was evaluated as the ratio of the indirect effect to the total effect (*b* = 0.21, *SE* = 7.69, *β* = 0.21, CI = [-0.15 – 22.78]), suggesting that 21% of the total effect of anxiety on difficulty concentrating is statistically attributable to worry in the specified model.

| **Table 2**  *Within- and between-person differences in DASS-21_Anx_ predicting PSWQ-PW (N = 197)* | | | | | | |
| --- | --- | --- | --- | --- | --- | --- |
|  | *b* | *β* | *SE* | *t* | χ^2^ (1) | *p* |
| Model 1 |  |  |  |  |  |  |
| Fixed effects |  |  |  |  |  |  |
| Intercept | 27.12 | 0.09 | 1.97 | 13.75 |  | < .001 |
| Time 2 | 1.71 | 0.08 | 0.83 | 2.07 |  | .039 |
| Time 3 | 1.30 | 0.06 | 0.84 | 1.56 |  | .120 |
| Participant mean DASS-21_Anx_ | 1.81 | 0.64 | 0.13 | 14.08 |  | < .001 |
| Random effects (*variance, SD*) |  |  |  |  |  |  |
| Participant (intercept) | 152.58 | 0.36 | 12.35 |  |  |  |
| Residual | 66.66 | 0.16 | 8.16 |  |  |  |
| Model 2 |  |  |  |  | 52.62 | < .001 |
| Fixed effects |  |  |  |  |  |  |
| Intercept | 27.14 | 0.09 | 1.97 | 13.79 |  | < .001 |
| Time 2 | 1.54 | 0.07 | 0.77 | 1.99 |  | .047 |
| Time 3 | 1.35 | 0.07 | 0.78 | 1.72 |  | .086 |
| Participant mean DASS-21_Anx_ | 1.81 | 0.65 | 0.13 | 14.08 |  | < .001 |
| Participant-centered DASS-21_Anx_ | 0.84 | 0.12 | 0.11 | 7.49 |  | < .001 |
| Random effects (*variance, SD*) |  |  |  |  |  |  |
| Participant (intercept) | 155.91 | 0.37 | 12.49 |  |  |  |
| Residual | 58.18 | 0.14 | 7.63 |  |  |  |
| *Note.* DASS-21_Anx_, Depression Anxiety Stress Scales 21 Anxiety composite; PSWQ-PW, Penn State Worry Questionnaire. | | | | | | |

| **Table 3**  *Within- and between-person differences in PSWQ-PW predicting ACS (N = 187)* | | | | | | |
| --- | --- | --- | --- | --- | --- | --- |
|  | *b* | *β* | *SE* | *t* | χ^2^ (1) | *p* |
| Model 1 |  |  |  |  |  |  |
| Fixed effects |  |  |  |  |  |  |
| Intercept | 39.66 | 0.03 | 1.64 | 24.13 |  | < .001 |
| Time 2 | -0.01 | -0.00 | 0.36 | -0.01 |  | .989 |
| Time 3 | 0.75 | 0.08 | 0.36 | 2.08 |  | .039 |
| Participant mean PSWQ-PW | 0.19 | 0.38 | 0.03 | 6.30 |  | < .001 |
| Random effects (*variance, SD*) |  |  |  |  |  |  |
| Participant (intercept) | 53.58 | 0.62 | 7.32 |  |  |  |
| Residual | 11.72 | 0.14 | 3.42 |  |  |  |
| Model 2 |  |  |  |  | 30.17 | < .001 |
| Fixed effects |  |  |  |  |  |  |
| Intercept | 39.84 | 0.05 | 1.64 | 24.29 |  | < .001 |
| Time 2 | -0.29 | -0.03 | 0.38 | -0.82 |  | .412 |
| Time 3 | 0.51 | 0.05 | 0.35 | 1.44 |  | .151 |
| Participant mean PSWQ-PW | 0.19 | 0.38 | 0.03 | 6.30 |  | < .001 |
| Participant-centered PSWQ-PW | 0.12 | 0.09 | 0.02 | 5.58 |  | < .001 |
| Random effects (*variance, SD*) |  |  |  |  |  |  |
| Participant | 53.73 | 0.62 | 7.33 |  |  |  |
| Residual | 10.83 | 0.13 | 3.29 |  |  |  |
| *Note.* PSWQ-PW, Penn State Worry Questionnaire – Past Week; ACS, Attentional Control Scale. | | | | | | |

| **Table 4**  *Sensitivity analyses with depression severity, sleep disturbance, and COVID-related worries as covariates* | | | | | | |
| --- | --- | --- | --- | --- | --- | --- |
|  | *b* | *β* | *SE* | *t* | *p* |  |
| Model 1 (predicting PSWQ-PW; *N* = 197) |  |  |  |  |  |  |
| Fixed effects |  |  |  |  |  |  |
| Intercept | 26.65 | 0.09 | 1.97 | 13.56 | < .001 |  |
| Time 2 | 1.45 | 0.07 | 0.77 | 1.89 | .059 |  |
| Time 3 | 1.31 | 0.06 | 0.78 | 1.69 | .092 |  |
| Participant mean DASS-21_Anx_ | 1.61 | 0.58 | 0.14 | 11.23 | < .001 |  |
| Participant-centered DASS-21_Anx_ | 0.70 | 0.02 | 0.12 | 5.82 | < .001 |  |
| DASS-21_Dep_ | 0.22 | 0.11 | 0.07 | 2.94 | .003 |  |
| Random effects (*variance, SD*) |  |  |  |  |  |  |
| Participant (intercept) | 154.55 | 0.36 | 12.43 |  |  |  |
| Residual | 57.31 | 0.13 | 7.57 |  |  |  |
| Model 2 (predicting ACS; *N* = 187) |  |  |  |  |  |  |
| Fixed effects |  |  |  |  |  |  |
| Intercept | 36.84 | 0.05 | 2.02 | 18.23 | < .001 |  |
| Time 2 | -0.32 | -0.03 | 0.34 | -0.94 | .346 |  |
| Time 3 | 0.46 | 0.05 | 0.35 | 1.32 | .189 |  |
| Participant mean PSWQ-PW | 0.14 | 0.29 | 0.03 | 4.61 | < .001 |  |
| Participant-centered PSWQ-PW | 0.10 | 0.07 | 0.02 | 4.33 | < .001 |  |
| DASS-21_Dep_ | 0.09 | 0.10 | 0.03 | 2.93 | .004 |  |
| PROMIS Sleep-SF | 0.15 | 0.03 | 0.07 | 2.23 | .027 |  |
| CRISIS Worries | 0.07 | 0.03 | 0.05 | 1.40 | .162 |  |
| Random effects (*variance, SD*) |  |  |  |  |  |  |
| Participant | 51.90 | 0.60 | 7.20 |  |  |  |
| Residual | 10.46 | 0.12 | 3.23 |  |  |  |
| *Note.* DASS-21_Anx_, Depression Anxiety Stress Scales 21 Anxiety composite; PSWQ-PW, Penn State Worry Questionnaire – Past Week; ACS, Attentional Control Scale; DASS-21_Dep_, Depression Anxiety Stress Scales 21 Depression subscale; PROMIS Sleep-SF, PROMIS Sleep Disturbance - Short Form; CRISIS Worries, Coronavirus Health Impact Survey COVID Worries Domain. | | | | | |  |

**Figure 1 – Mediation models.** Structural equation model with worry as a mediator of the association between anxiety and difficulty concentrating, without covariates (*N* = 184). Coefficients are standardized betas.


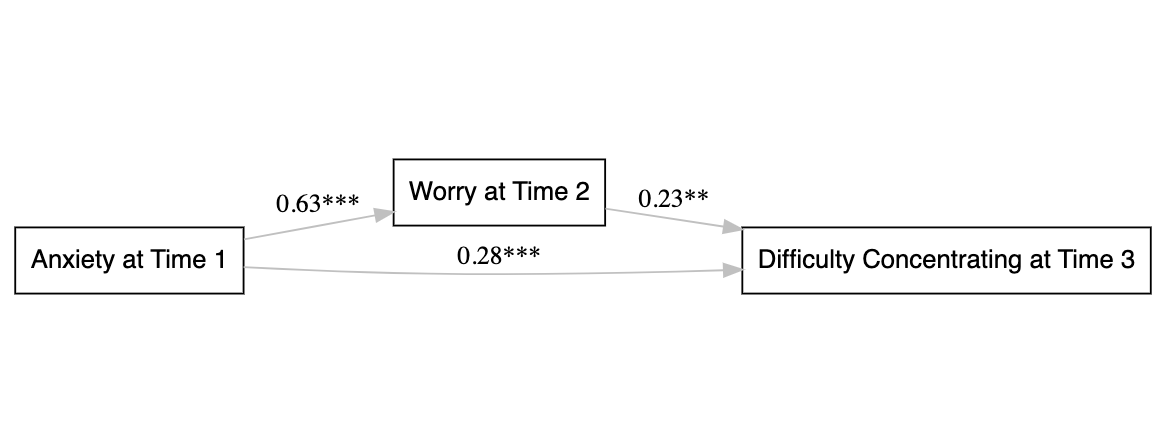


**Figure 2 – Mediation model with covariates.** Structural equation model with worry as a mediator of the association between anxiety and difficulty concentrating, regressing each outcome on itself at the previous timepoint (*N* = 180). Coefficients are standardized betas. All paths are estimated simultaneously so path coefficients represent the effect of each predictor on the linked outcome controlling for all other predictors linked to that outcome.


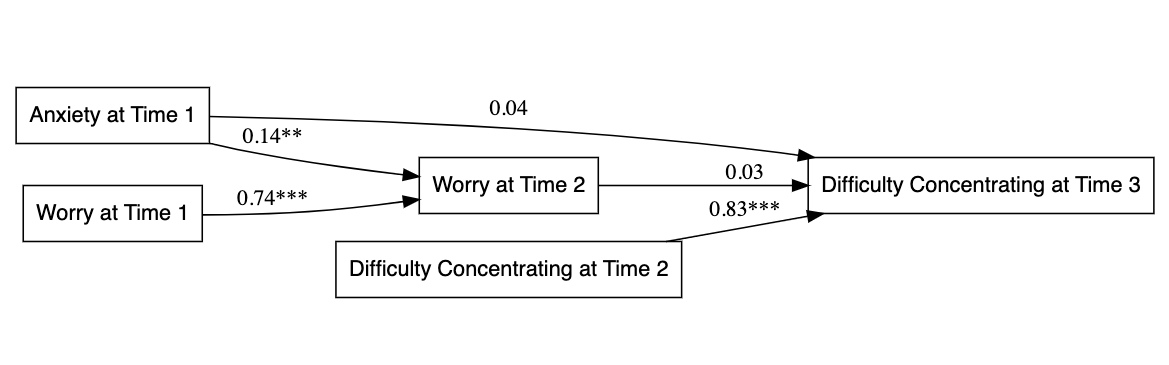


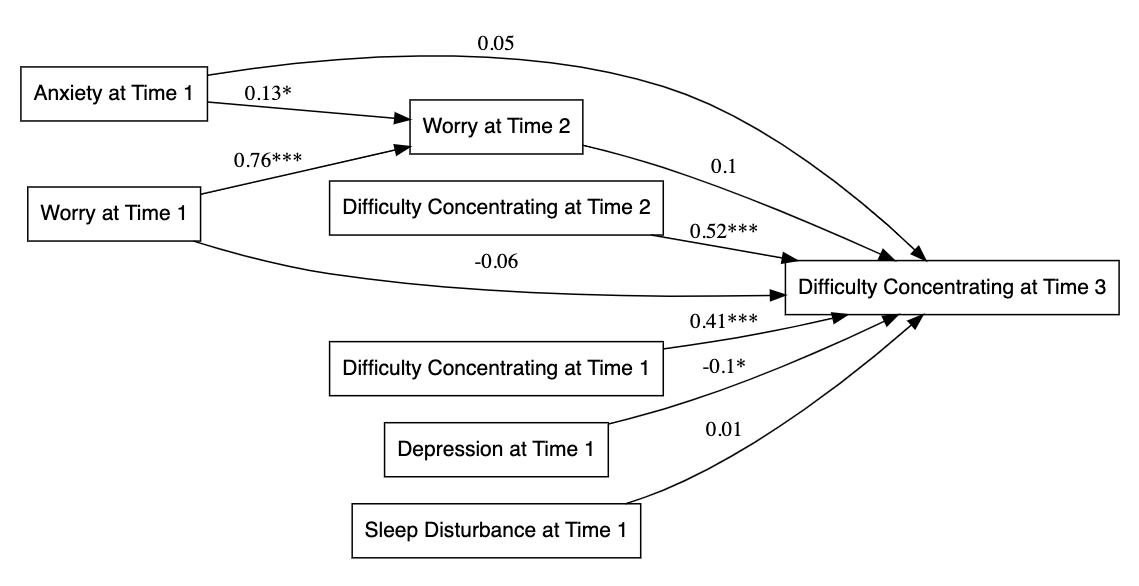
**Figure 3 – Mediation model with full covariates.** Structural equation model with worry as a mediator of the association between anxiety and difficulty concentrating, regressing each outcome on itself at the previous timepoint, and including depression, sleep disturbance, and difficulty concentrating at T1 as additional exogenous predictors of difficulty concentrating at T3 (*N* = 175). Coefficients are standardized betas. All paths are estimated simultaneously so path coefficients represent the effect of each predictor on the linked outcome controlling for all other predictors linked to that outcome.
